# Phospholipid profiling of plasma from GW veterans and rodent models to identify potential biomarkers of Gulf War Illness

**DOI:** 10.1371/journal.pone.0176634

**Published:** 2017-04-28

**Authors:** Tanja Emmerich, Zuchra Zakirova, Nancy Klimas, Kimberly Sullivan, Ashok K. Shetty, James E. Evans, Ghania Ait-Ghezala, Gary S. Laco, Bharathi Hattiangady, Geetha A. Shetty, Michael Mullan, Gogce Crynen, Laila Abdullah, Fiona Crawford

**Affiliations:** 1 The Roskamp Institute, Sarasota, Florida, United States of America; 2 The Open University, Milton Keynes, Buckinghamshire, United Kingdom; 3 James A. Haley Veteran’s Hospital, Tampa, Florida, United States of America; 4 NOVA Southeastern University, Ft. Lauderdale and Miami VAMC, Miami, Florida, United States of America; 5 Boston University School of Public Health, Boston, Massachusetts, United States of America; 6 Research Service, Olin E. Teague Veterans’ Medical Center, Temple, Texas, United States of America; 7 Institute for Regenerative Medicine, Texas A&M University College of Medicine, College Station, Texas, United States of America; Technion Israel Institute of Technology, ISRAEL

## Abstract

Gulf War Illness (GWI), which affects at least one fourth of the 700,000 veterans deployed to the Gulf War (GW), is characterized by persistent and heterogeneous symptoms, including pain, fatigue and cognitive problems. As a consequence, this illness remains difficult to diagnose. Rodent models have been shown to exhibit different symptomatic features of GWI following exposure to particular GW agents (e.g. pyridostigmine bromide, permethrin and DEET) and/or stress. Preclinical analyses have shown the activation of microglia and astroglia as a pathological hallmark in these mouse and rat models. Although much has been learned in recent years from these different rodent models and independent clinical studies, characterization studies to identify overlapping features of GWI in animals and humans have been missing. Thus, we aimed to identify biomarkers that co-occur in the plasma of rodent models of GWI and human GWI patients. We observed increases of multiple phospholipid (PL) species across all studied cohorts. Furthermore, these data suggested dysfunction within ether and docosahexaenoic acid and arachidonic acid containing PL species in relation to GWI. As these PL species play a role in inflammatory processes, these findings suggest a possible role for inflammatory imbalance in GWI. Overall, we show that the peripheral lipid disturbances are present both in human GWI patients and in the preclinical rodent models of GWI, highlighting the importance of lipidomics as a potential platform for further biomarker discovery and supporting the value of GW agent exposed models of GWI.

## Introduction

Gulf War Illness (GWI) is a chronic multisymptom illness, which affects approximately one fourth of the 700,000 US veterans, who were deployed to the 1990–91 Persian Gulf War (GW) [1–7]. Veterans with GWI experience chronic health symptoms such as fatigue, muscle and joint pain and gastrointestinal problems [8]. Among the central nervous system (CNS) based symptoms, memory problems are among the most commonly reported complaints [1,2,5,8–13]. The multiplicity and heterogeneity of symptoms observed in GW veterans is unique to the 1990–91 deployment, with no identical illness being reported in any other military campaign, indicating that GWI etiology cannot solely be attributed to combat-related stress [2,10,14–17]. Twenty-five years later, GW veterans are still struggling with this chronic illness, which remains difficult to diagnose due to a lack of objective diagnostic tests. Currently, clinical diagnosis is most commonly made by self-report of health symptoms using the Fukuda Center for Disease Control (CDC) or the Kansas criteria [2,5,8,18]. Consequently, many GW veterans report symptoms consistent with GWI but have not received a formal diagnosis of their condition. Blood based biomarkers of GWI are needed to assist clinicians with providing an objective diagnosis of GWI and for developing targeted treatment strategies.

After performing a comprehensive review of clinical and animal research studies conducted to identify the causes of GWI, the Research Advisory Committee (RAC) on GW Veterans’ Illness concluded that the key contributors to the etiology of GWI included combined war-time exposure of the prophylactic anti-nerve gas pill pyridostigmine bromide (PB) and pesticides which were also used prophylactically to protect against insect-borne diseases [1,7,8]. The many pesticides used during the GW included irreversible and reversible acetylcholinesterase inhibitors (AChEi) including organophosphate (OP) and carbamate pesticides [1,8]. Other commonly used pesticides included pyrethroids, such as permethrin (PER), the insect repellant N, N-diethyl-m-toluamide (DEET) and organophosphates such as diisopropyl fluorophosphate (DFP) [1,14–16,19–23]. Organophosphate exposure from low-level sarin nerve agent exposure also occurred in many GW veterans and is modeled in animal studies with the sarin surrogate diisopropyl fluorophosphate (DFP). Results of these studies showed a chronic neuroinflammatory phenotype as a result of this sarin-surrogate exposure, particularly when administered in conjunction with a physical stressor [24].

The complex clinical presentation of GWI suggests that various biological and metabolic processes might be altered in GW veterans. For example, impaired immune responses have been observed in veterans with GWI [8]. Veterans with GWI showed altered expression of pro-and anti-inflammatory cytokines on their peripheral immune cell. Additionally, immune system irregularities were especially pronounced in GWI patients compared to controls during exercise challenge, such as increased IL-6, IL-10, and TNF-α levels, as well as a Th1/Th17 immune polarization [25–30].

Phospholipids (PL) are critical components of most cellular membranes, including mitochondrial membranes, and their metabolism generates bioactive lipids that can modulate inflammatory pathways [31]. In particular, phosphatidylcholine (PC) and phosphatidylethanolamine (PE) serve as a reservoir for ω-3 docosahexaenoic acid (DHA) and ω-6 arachidonic acid (AA), metabolism of which generates bioactive lipid that can alter the immune/inflammatory responses in both the central and the peripheral nervous system. For instance, DHA is a precursor of the anti-inflammatory mediators, resolvins and neuroprotectins, whereas AA is a precursor of some pro-inflammatory lipid mediators, such as prostaglandins and leukotrienes [32,33]. Preclinical studies have shown that exposure to GW agents can affect the brain phosphocholine levels [34], which is a metabolite of PC required for the endogenous synthesis of acetylcholine (ACh) [35]. In addition, mitochondrial dysfunction is thought to underlie the clinical features of GWI [36–38]. Furthermore, mitochondria also contain substantial amounts of PL, of which 75% are PC and PE and 10% are phosphatidylinositol (PI) [39].

We previously established several mouse models using exposure to different combinations of these GW agents to investigate the complex underlying GWI pathology, which presumably involves multiple physiological and biochemical mechanisms. We showed that PB+PER co-administration in a CD1 mouse model daily for 10 consecutive days led to observed anxiety 30 days post exposure and delayed cognitive impairment 5 months post exposure [16]. Additionally, at 5 months post exposure, astrogliosis was observed in exposed mice. Proteomic analyses revealed alteration of multiple biological systems, such as endocrine function, immune and inflammatory pathways, as well as disturbances in lipid metabolism. For example, the fatty acid-binding protein 3 (FABP3) was reduced in exposed animals [16]. This protein plays an important role in the uptake of AA in the brain [40]. We subsequently translated the PB+PER exposure model to the more commonly used C57BL6/J strain of mice, again showing cognitive deficits associated with increased astrogliosis, as well as reduction of synaptophysin staining in the hippocampi and cerebral cortices at 5 months post exposure [41]. Since the neurobehavioral features we observed in our mouse model correlate with symptoms that are relevant to the clinical presentation of GWI, it is possible that identified pathologies in these animals are also present in human GWI patients [5,9,42]. We have also reported elevated ether and diacyl PC species in the brains of these mice [34].

The insect repellant, DEET has also been named one of the key contributors to GWI, and it has been suggested that stress modulates GWI symptoms in humans [6,42,43]. Addressing these additional exposures, Shetty and colleagues developed and characterized a rat model of GWI using a PB+PER+DEET exposure paradigm, along with 5 min of restraint stress to mimic war related stress [14,44,45]. Their rat GWI model showed memory and mood dysfunction evident by deficits in spatial memory and increased depressive behavior [44]. They also observed some loss of glutamatergic neurons, activated microglia, hypertrophy of astrocytes, reduced neurogenesis [44] and loss of certain subpopulations of gamma-amino butyric acid (GABA)-ergic interneurons in the hippocampus [46].

These mouse and rat studies have been critical for advancing our understanding of the different facets of GWI pathology and for characterizing the underlying biological features. However, translational studies between different animal models and human patients are currently lacking. Furthermore, there remains a need for identifying novel biomarkers, which can assist with diagnosing GWI and may also help link preclinical and clinical studies aimed at developing therapies. We deliberately chose two different models of GWI to capture the diversity of exposure paradigms experienced by the soldiers during the Gulf War. Taking into account the role of PL disturbances detected in preclinical rodent models of GWI and the need for translational validation studies, we hypothesize that exposure to GW chemicals in rodents may correspond with changes in plasma PL profiles. We also propose that overlapping changes in PL profiles between diverse rodent models of GWI may capture aspects of PL abnormalities in GW veterans with this multisymptom illness, which could aid with diagnosing this illness in the future.

We performed lipidomic analysis in the plasma of two different rodent models described above, which we then compared to results from a cohort of GW veterans with and without GWI.

## Materials & methods

### Animals

#### GWI mouse model

All mouse experiments were conducted at the Roskamp Institute in Sarasota, Florida and were approved by the Roskamp Institute’s Institutional Animal Care and Use Committee and conducted in accordance with the Office of Laboratory Animal Welfare and the Association for the Assessment and Accreditation of Laboratory Animal Care. Mice were purchased from Jackson Laboratories (Bar Harbor, Maine) and each mouse was individually housed in a controlled environment (regulated 14-h day/10-h night cycle) and maintained on a standard diet. We used a standard diet (ENVIGO, IN) that contains a standard mixture of macronutrients (protein (19.1%), fat (6.5%), carbohydrate (47%), crude fiber (2.7%), neutral detergent fiber (12.3%), ash (5.1%)) and fatty acids (both omega-6 and omega-3, SFA (0.8%), MUFA (1.1%) and PUFA (2.9%)). The diet contained 2.6% linoleic acid and 0.3% alpha linolenic acid and contained less than 0.01% arachidonic acid (AA), docosahexaenoic acid (DHA) and eicosapentaenoic acid (EPA).

Since control animals are on the same diet, any potential dietary issues are controlled for in this study design. The exposure paradigm has been previously described [41,47]. Male C57BL6/J mice (12 weeks of age) were co-administered with either a 50μl total volume of GW agents to a final dose of 0.7 mg/kg of PB (Fisher Scientific (Hanover Park, IL)) and 200 mg/kg of PER (Sigma Aldrich (St. Louis, MO)) in 100% dimethyl sulfoxide (DMSO) [exposed mice; n = 4], or a 50μl volume of vehicle (100% DMSO) [sham mice; n = 4] via intraperitoneal injection (IP) injection daily, for 10 days. According to the RAND survey, PB consumption was to commence only after the commander’s order and, as such, not all soldiers took PB throughout the conflict. It is unclear when the orders to take PB were issued in relation to the actual battle. The report also suggests that tablets were taken during the ground war, which only lasted days. As our focus was co-exposure to pesticides and PB over a short-term so an acute co-exposure to PB and PER was administered for 10 days as the basis for mouse model development [48]. The dose of 0.7mg/kg for PB was utilized based on the estimates provided by the Department of Defense suggesting that a number of military personnel took two 30 mg tablets (60mg/75kg per person equals about 0.8mg/kg) per day during the GW [49] and was determined to be suitable for administration in C57BL6 mouse strain based on our pilot work. There is no accurate account of how much PER was used during the GW as soldiers liberally used these chemicals where it was applied on the skin surface directly, inhaled through sprays and was embedded in their uniforms [1]. Therefore, we used 200 mg/kg of PER in order mimic a high-level exposure, which has shown adverse pathological outcome in previous studies [34,41,47,50–53]. The doses of PB and PER are less than one fifth and one half of the known LD50 values in rodents, respectively [54,55]. Six months post exposure, mice were euthanized, and plasma was collected. Mice were exsanguinated via cardiac puncture using an 18-gauge wide-bore needle to prevent hemolysis of red blood cells (RBC) during blood collection. Blood samples were collected into a 1.5 ml Eppendorf tube containing 10 units of heparin and a protease inhibitor cocktail (Roche, NJ) to a final concentration of 1 x. Samples were immediately centrifuged at 3000 x g for 5 min and the plasma was transferred to a new 1.5 ml Eppendorf tube and snap frozen in liquid nitrogen. Plasma samples were stored at -80C until further biochemical studies.

#### GWI rat model

All rat studies were conducted at the Central Texas Veterans Healthcare System in Temple, TX. Specifically, Sprague-Dawley rats were exposed to PB, DEET, PER and stress using previously described procedures developed by Shetty et al. [44,45]. Experiments were in compliance with the National Institutes of Health guidelines for care and use of animals and in accordance with the animal protocol approved by the animal care and use committee of the Central Texas Veterans Health Care System, Temple TX. Three-month-old male rats were obtained from Harlan (Indianapolis, IN) and housed for 2 weeks before being exposed to GW agents. All rats were given food (commercial rat chow) and water *ad libitum*. The rats diet (commercial rat chow; PicoLab Rodent Diet 20) contained macronutrients ((protein (20%), fat (4.5%), carbohydrate (52.9%), crude fiber (7%) and ash (7%)) as well as fatty acids (both omega-6/omega-3, SFA (0.93%), MUFA (0.99%) and PUFA (3.6%) normally provided in any rodent diet. The relative proportion of SFA to MUFA to PUFA was similar in the two rodent models of GWI. Furthermore, the diet was composed of 2.19% linoleic acid and 0.26% alpha linolenic acid and contained less than 0.01% of AA, DHA and EPA.

EPA

As with mice, control and exposed rats were on the same diet, and therefore any potential dietary issues were controlled for in this study design.

Although the ground war itself was short, the bombing continued for several weeks and it is also suggested that solders may have taken PB throughout the conflict. In contrast to the described mouse model above, the 4-week exposure timeframe was used in the rat model to match a potential long-term exposure of veterans to these GW agents.

The exposure paradigm included PB (1.3 mg/kg; Sigma, St. Louis, MO), PER (200 μl, 0.13 mg/kg in 70% alcohol; Chem. Service Inc., West Chester, PA), DEET (200 μl containing 40mg/kg in 70% alcohol; Chem. Service Inc., West Chester, PA), as well as 5 min of daily restraint stress, via a rat restrainer for 4 weeks (exposed rats n = 5). PB was administered through oral gavage, which corresponds to the veteran population that were given a course of twenty-one 30-mg tablets of PB every 8 hours as prophylaxis against organophosphate nerve agents [56]. This leads to a total consumption of 120mg/kg per day. For an average weight of 75 kg per individual this would approximate 1.6 mg/kg. Rats were given 1.3mg/kg. Application of pesticides in this model was limited to dermal exposure route so PER and DEET solutions were applied to shaved skin areas located on the back of the neck. Non-exposed sham rats were naïve controls (n = 6). Plasma samples were collected 6 months post exposure to GW agents and stress and were sent to the Roskamp Institute investigators on dry ice.

### Human subjects

Plasma samples from veterans, deployed to the 1990–1991 GW, were provided by the Boston University and the Nova Southeastern University investigators from an established biorepository of GW veterans, who agreed to share their blood samples from prior studies. This includes the Boston Gulf War Illness Consortium (GWIC) and the Dynamic Modeling of GWI study from Nova Southeastern University and the CDMRP funded study to the South Florida Veterans Affairs Foundation for Research and Education, Inc. The GWI biorepository is approved by the IRBs from Boston University, Nova Southeastern University and the Miami VAMC. Samples from the GWI biorepository were all collected from the Boston and Miami areas using the same standard operating procedures for phlebotomy, plasma separation and aliquoting. Plasma samples were obtained from fasting subjects. All samples were stored at -80°C and were not previously thawed and refrozen. GW veteran participants were consented into their respective studies using ICH GCP guidelines. The Kansas GWI criteria [5] were used to determine cases of GWI and controls. The Kansas GWI criteria require that GW veterans endorse symptoms in at least 3 of 6 symptom domains (fatigue/sleep problems, pain, cognitive, mood symptoms, gastrointestinal symptoms, respiratory symptoms and skin abnormalities). Controls were deployed veterans from the 1991 GW who did not meet the Kansas GWI criteria. Subjects also completed demographics and health symptom questionnaires including the Pittsburgh Sleep Quality Index (PSQI), Visual Analog Scale (VAS) for pain, Multi-dimensional Fatigue Inventory (MFI-20) questionnaire, MOS Short Form 36-veteran version (SF-36V), and Profile of Mood States (POMS). Study participants were excluded if they reported being diagnosed with another medical condition that could explain the above mentioned symptoms according to the Kansas GWI case definition exclusions, including veterans with a history of prior central nervous system or major psychiatric disorders that may affect cognitive function (e.g., epilepsy, stroke, brain tumor, multiple sclerosis, Parkinson’s Disease, Alzheimer’s disease, schizophrenia). Control veterans (n = 11) and veterans with GWI (n = 22) were matched for age, gender and ethnicity. Due to low numbers in the different ethnic groups, we dichotomized the ethnicity into Caucasian (n = 17) vs. non- Caucasian (n = 16) in order to study ethnicity effect on PL levels in the entire cohort population independently of diagnosis. The non-Caucasian group included African American (n = 8), Hispanic (n = 4), Asians (n = 2) and others (n = 2). Baseline demographics can be found in Table 1.

**Table 1:** Baseline demographics of Gulf War veteran cohort.

	Control	GWI
**N total**	11	22
**Age (Mean±Stdev)**	48.5±7.7	48.4±6.3
**Male (%)**	9 (81.9%)	17 (77.3%)
**Ethnicity**		
*Caucasian*	5	12
*African American*	3	5
*Hispanic*	2	2
*Asian*	0	2
*Other*	1	1

### Lipidomic analysis

Lipidomic analyses were performed as previously described (Emmerich et al., 2015). Briefly, the experimenter was kept blinded to the diagnostic classification of the samples from the clinical cohort and the exposure assignment of samples from the animal studies. Lipids were extracted from plasma of human, rat and mice samples using the Folch method [57]. Synthetic internal standards (di-14:0 fatty acid [FA] containing PC and PE, 14:0 FA containing LPE and LPC, and di-16:0 for phosphatidylinositol [PI]) were added to plasma prior to lipid extraction. As previously described [58], lipid extracts were resuspended in isopropanol and separation was achieved using hydrophilic interaction chromatography (HILIC) on a 1mm x 100mm column packed with 3μm Pinnacle II silica particles (Restek Corporation, Bellefonte, PA, USA). Two reference samples for each cohort were extracted together with the rodent and human samples and added to each run for analytical quality control. A fifteen minute isocratic separation was performed with 70% solvent A (100% acetonitrile [ACN]) and 30% solvent B (78% methanol, 1% formic acid, 0.6% ammonium hydroxide) at a flow rate of 55 μl/min with the column temperature at 40°C. Mass spectrometry (MS) was performed with a Thermo LTQ-XL linear ion trap mass spectrometer equipped with a Surveyor HPLC pumping system and Micro AS autosampler (Thermo-Fisher, Waltham, MA, USA). Full scan negative ion electrospray mass spectra were acquired from m/z 200 to 2,000 with in-source collision induced dissociation (SCID), with relative energy of 15%. All spectra were obtained with a 200 msec maximum ion time and by summing of 5 microscans. Mass spectra were summed over the chromatographic peak for each PL class, limited to values above a threshold of 0.01% base ion intensity, exported to Microsoft Excel (Microsoft, Redmond, WA, USA), and then analyzed with LipidomeDB online using custom target lists for each PL class (University of Kansas, Lawrence, KS, USA; http://129.237.137.125:8080/Lipidomics/) to identify and quantify each PL molecular species.

The measured concentration of all molecular species identified within each PL class were summed to generate total PC, LPC, PE, LPE and PI concentration values. Each phospholipid class of PC, LPC, PE, LPE, and PI was then separately grouped according to their degree of unsaturation of each molecular species (SFA—saturated fatty acids, MUFA—monounsaturated fatty acids, and PUFA—polyunsaturated fatty acids). Sphingomyelin was excluded from this analysis due to its lack of PUFA-containing SM species.

Within each class, we grouped arachidonic acid (AA) containing lipid species and docosahexaenoic acid (DHA) containing species of PC, LPC, PE, LPE and PI as previously described [59]. We previously determined the identification of these species as containing AA and DHA based on MS/MS analyses [60]. Ether PC (ePC), ePE, eLPC and eLPE were grouped separately, as these lipids contain ether linkage at the SN1 position.

### Statistical analyses

Data were checked for normal distribution histogram plots and skewness/kurtosis measures. Statistical analyses were then performed as we have previously described [52,58]. Principal Component Analysis (PCA) was used to minimize multi-colinearity and achieve dimension reduction, as used routinely for the evaluation of lipidomic data. First, the Kaiser-Mayer-Olkin (KMO) and Bartlett’s test for Sphericity was used to ensure sampling adequacy for PCA. Sampling adequacy as determined by a KMO value of > 0.6 and Bartlett p value < 0.05 were further investigated. Variables with eigenvalues of ≥1 were retained, PCA was used for extracting components, and varimax with Kaiser normalization was used for rotation to simplify and clarify the data structure. In order to perform mixed linear modeling (MLM) regression analysis on each component (the outcome measure), the Anderson-Rubin method was used for exporting uncorrelated scores while adjusting for random (mice) and fixed (injury, time and replication) factors. Following data analysis using MLM, Fisher's least significant difference (LSD) correction and the Benjamini–Hochberg procedure (B-H) were used for multiple-test correction and control of false discovery rate (FDR) for all comparisons (total lipids, ether lipids, DHA and AA). All data were analyzed using SPSS version 22.0.0 (IBM Corporation, Armonk, NY). B-H (α = 0.05) was calculated using JMP 11 (SAS, Cary, NC

## Results

Our aim was to first establish a plasma PL profile that could distinguish veterans with GWI from deployed controls. We examined changes in total PL levels, (PC, PE, PI, LPC and LPE), as well as the degree of unsaturation of PL class, ether content and ω-3 and ω-6 fatty acid (FA) composition. We then investigated the association of individual PL molecular species with the diagnosis of GWI. Finally, we examined these species in rodent models of GWI in order to be able to validate these preclinical models.

Results of principal component analysis can be found in the S1–S3 Tables. Data were normally distributed and as such are presented as means and standard error of the mean (SEM), as well as in μM ± SEM in S4–S7 Tables.

### Comparison of total phospholipid classes in GWI patients and GW deployed controls

Our case control cohort included a total of 33 veterans who were deployed to the GW region during 1990–1991; 11 were classified as controls and 22 veterans were diagnosed with GWI. In comparing baseline characteristics, control and GWI veterans were similar for gender, ethnicity and age (see Table 1). In order to determine if gender, age and ethnicity could potentially be confounding factors on the relationship between GWI and PL, we examined their influence on PL levels in the entire cohorts (combined controls and GWI). A gender effect was only observed for total PE (p = 0.004) levels. There was no significant influence of ethnicity for any of the PL classes. There was a significant correlation of age with LPC (r = 0.49; p<0.001) and PI (r = 0.27; p = 0.005).

**Fig 1:**
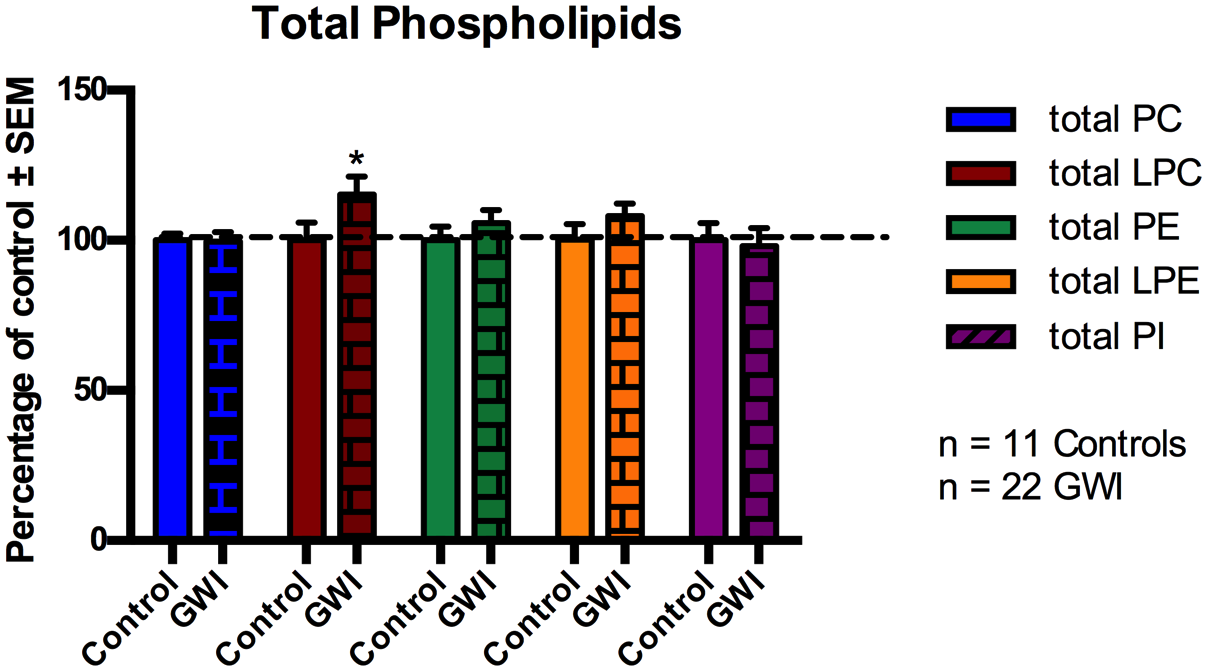
Changes in total plasma phospholipid levels in GWI subjects and controls, represented in mean ± SEM in percentage of control. Individual molecular species for each PL class identified by LC/MS were summed to calculate total PL levels within each class. *p < 0.05; MLM regression with *post hoc* analysis. Total LPC was significantly increased in GWI subjects (brick bars) compared to GV controls (solid bars).

We investigated changes in total PL levels for GWI cases compared to controls, presented as a percentage of control and shown in Fig 1 (Fig 1). For all subsequent analyses, we used gender as a covariate for all analyses pertaining to PE and age as a covariate for PI and LPC in order adjust for their potential confounding effects on relevant PL class. There were no significant differences for total PC, PE, LPE and PI between the two comparison groups (p>0.05). However, for total LPC post-hoc analysis revealed a significant increase of 15% for GWI patients versus controls (p = 0.020).

### Analysis of the degree of unsaturation of PL classes in human subjects

**Fig 2:**
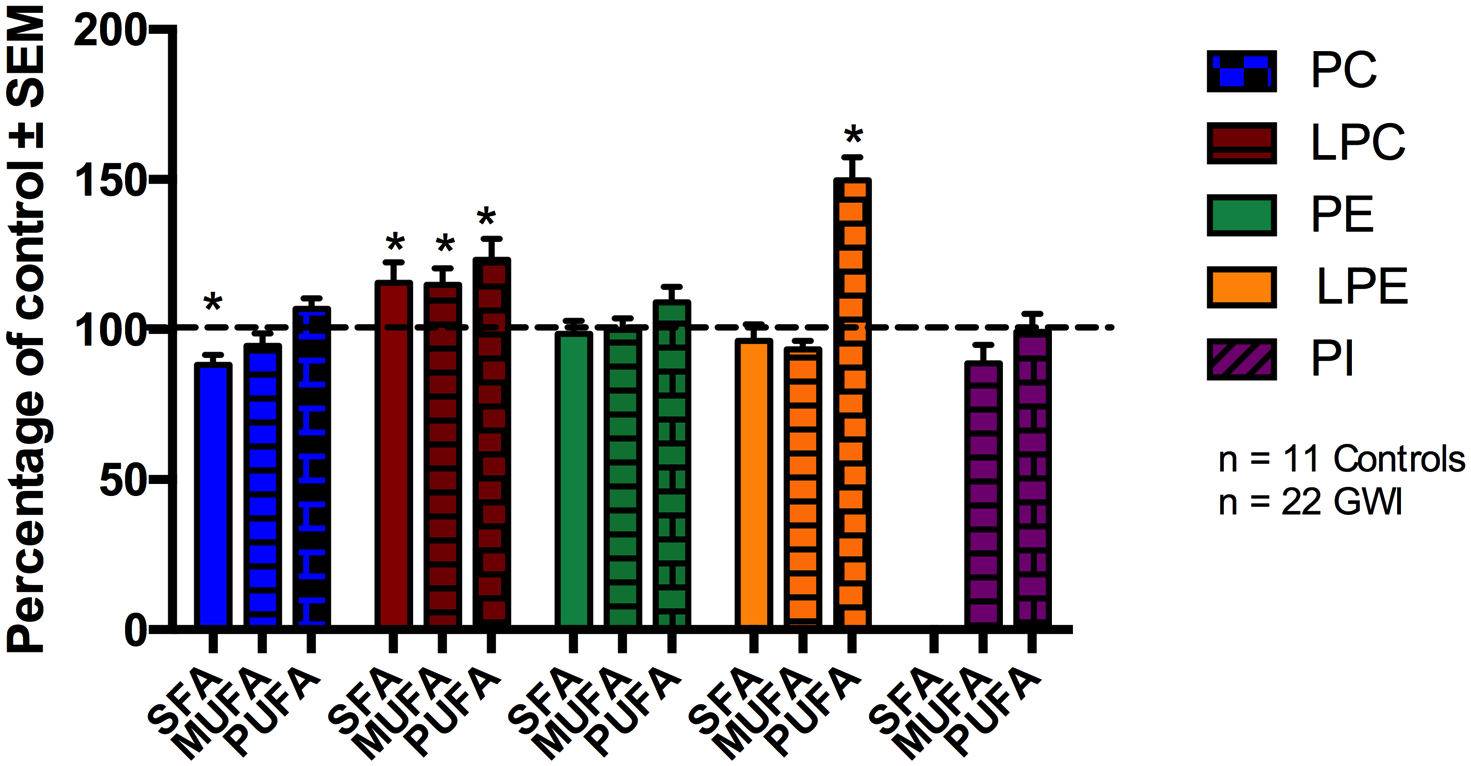
Degree of unsaturation of PL classes in plasma of GWI patients compared to controls, represented in mean ± SEM in percentage of control. For PC, SFA containing PC species were decreased in GWI patients, whereas for LPC and LPE, PUFA containing species were elevated compared to control subjects. No differences in the degree of unsaturation were observed for LPC and PE. For PI, no SFA containing species were identified (SFA: solid bars, MUFA: stripped bars, PUFA: brick bars). *p<0.05; MLM regression with *post hoc* analysis.

It has been shown that the brain can synthesize SFA and MUFA, however most essential PUFA are acquired through the periphery due to the low capacity of the brain to synthesize these *de novo* [61]. In order to investigate if GWI diagnosis influences unsaturation status of different PL classes, we examined SFA, MUFA and PUFA containing PL in human subjects. Fig 2 (Fig 2) shows SFA, MUFA and PUFA containing species of PC, LPC, PE, LPE and PI in GWI subjects as a percentage of controls. For PI, no SFA-containing species were found.

There were no differences between veterans with GWI and controls for the degree of unsaturation for PE and PI even after adjusting for potential confounding by gender and age, respectively. However, SFA containing PC species were reduced by 22% in GWI patients compared to controls (p = 0.024). No changes were seen in MUFA and PUFA containing PC species. In addition, the LPC, SFA, MUFA, and PUFA containing species were significantly elevated in GWI compared to controls by 16%, 15% and 23%, respectively, and remained significant after adjusting for age (p < 0.05). For LPE, PUFA containing species showed an increase of 50% in GWI compared to controls (p<0.001), whereas SFA and MUFA containing LPE species did not differ between the two groups.

### Examination of ether lipids in plasma of GWI cases compared to controls

**Fig 3:**
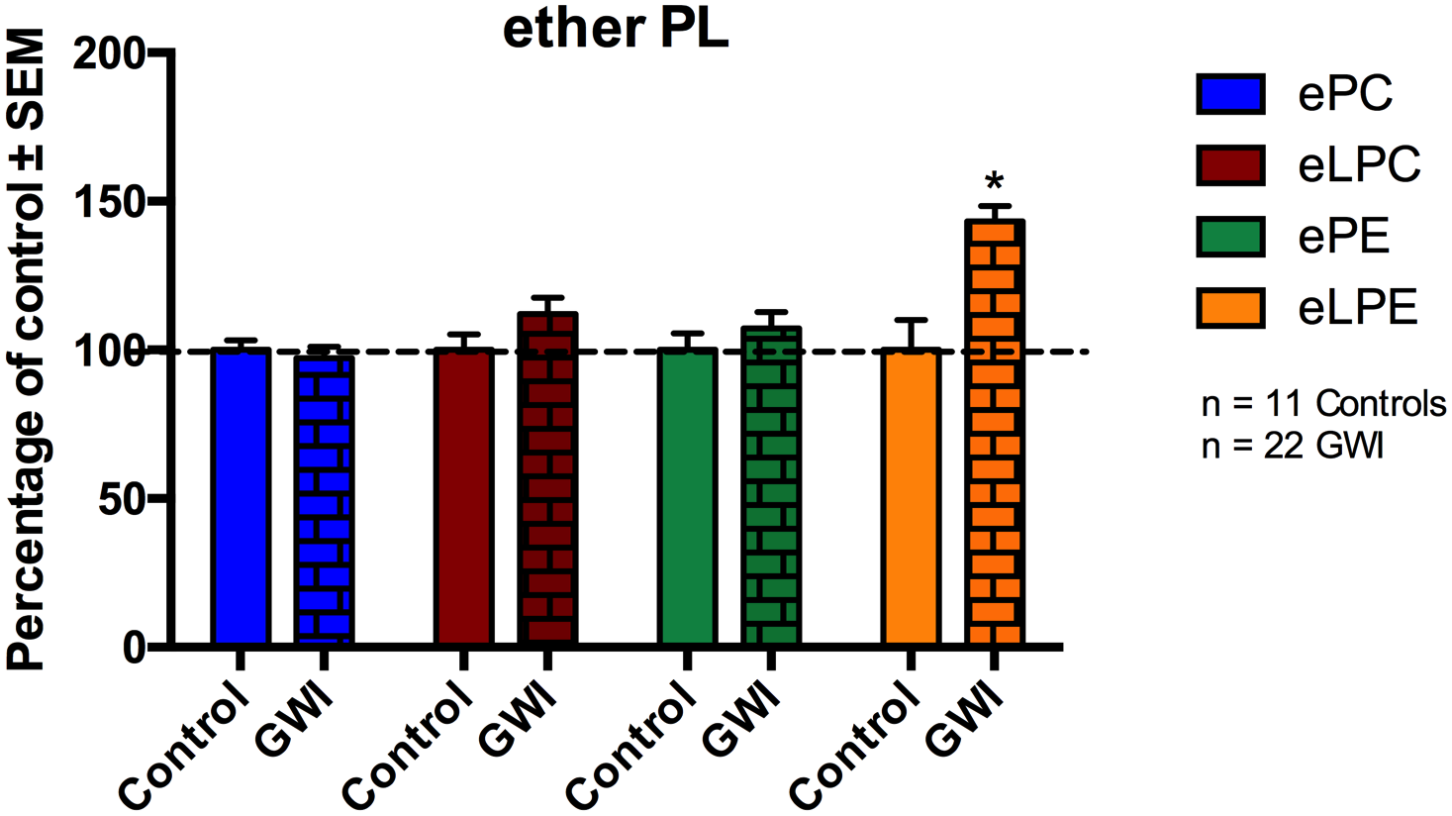
Ether lipid changes in plasma of GWI patients and compared to controls, represented in mean ± SEM in percentage of control. Levels of eLPE were significantly elevated in GWI patients (brick bars) compared to healthy GW controls (solid bars). No differences were observed for ePC, eLPC and ePE. *p<0.05; MLM regression with *post hoc* analysis.

Ether PL are dependent upon peroxisomes for their synthesis [62]. Therefore, we grouped ether PL within each class. Fig 3 (Fig 3) shows levels of ether PC, LPC, PE and LPE in GWI subjects compared to control. No differences were observed for ePC, eLPC and ePE between GWI and control subjects. However, eLPE levels were increased in GWI veterans by 43% relative to controls (p<0.001).

### Profiling of AA- and DHA-containing phospholipid species in GWI cases

Arachidonic acid (ω-6) and DHA (ω -3) containing fatty acids play a potential role in modulating inflammatory responses. Thus, individual PL species of the different classes were summed together if they had an AA containing fatty acid chain or DHA fatty acid chain respectively.

**Fig 4:**
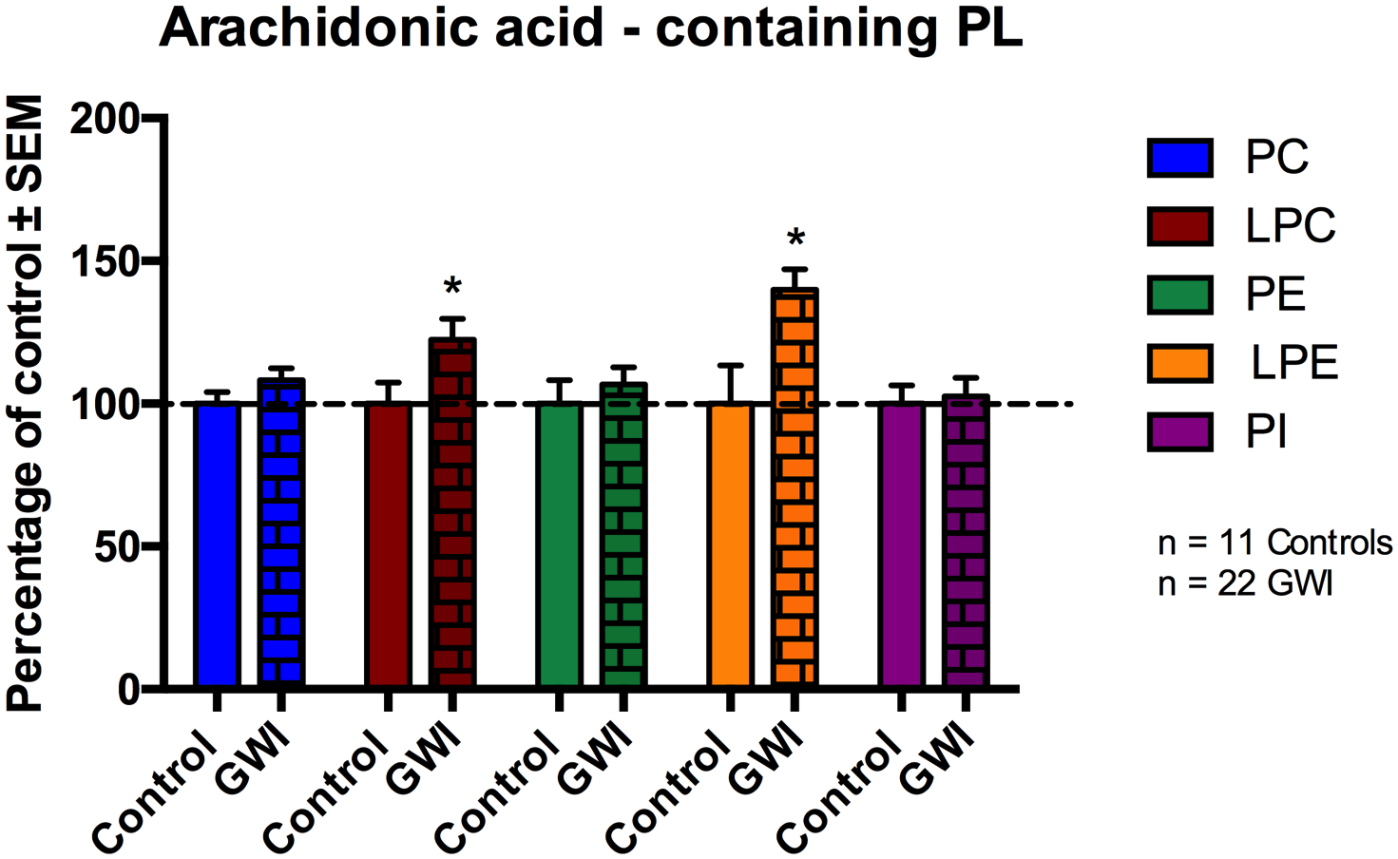
AA containing phospholipid species in plasma of GWI patients compared to controls, represented in mean ± SEM in percentage of control. Within GWI patients (brick bars), AA containing LPC and LPE species were significantly elevated compared to controls. No differences were observed within PC, PE and PI. *p<0.05; MLM regression with *post hoc* analysis.

Fig 4 (Fig 4) shows AA containing PL species of PC, LPC, PE, LPE and PI. No differences were observed for AA containing PC, PE and PI between the two groups. Compared to controls, AA species within LPC and LPE were increased by 22% (p = 0.023) and 40% respectively (p = 0.005; for LPC the p value was adjusted for potential confounding by age) in GWI compared to control subjects. For LPE, age was not detected as a cofounding factor.

**Fig 5:**
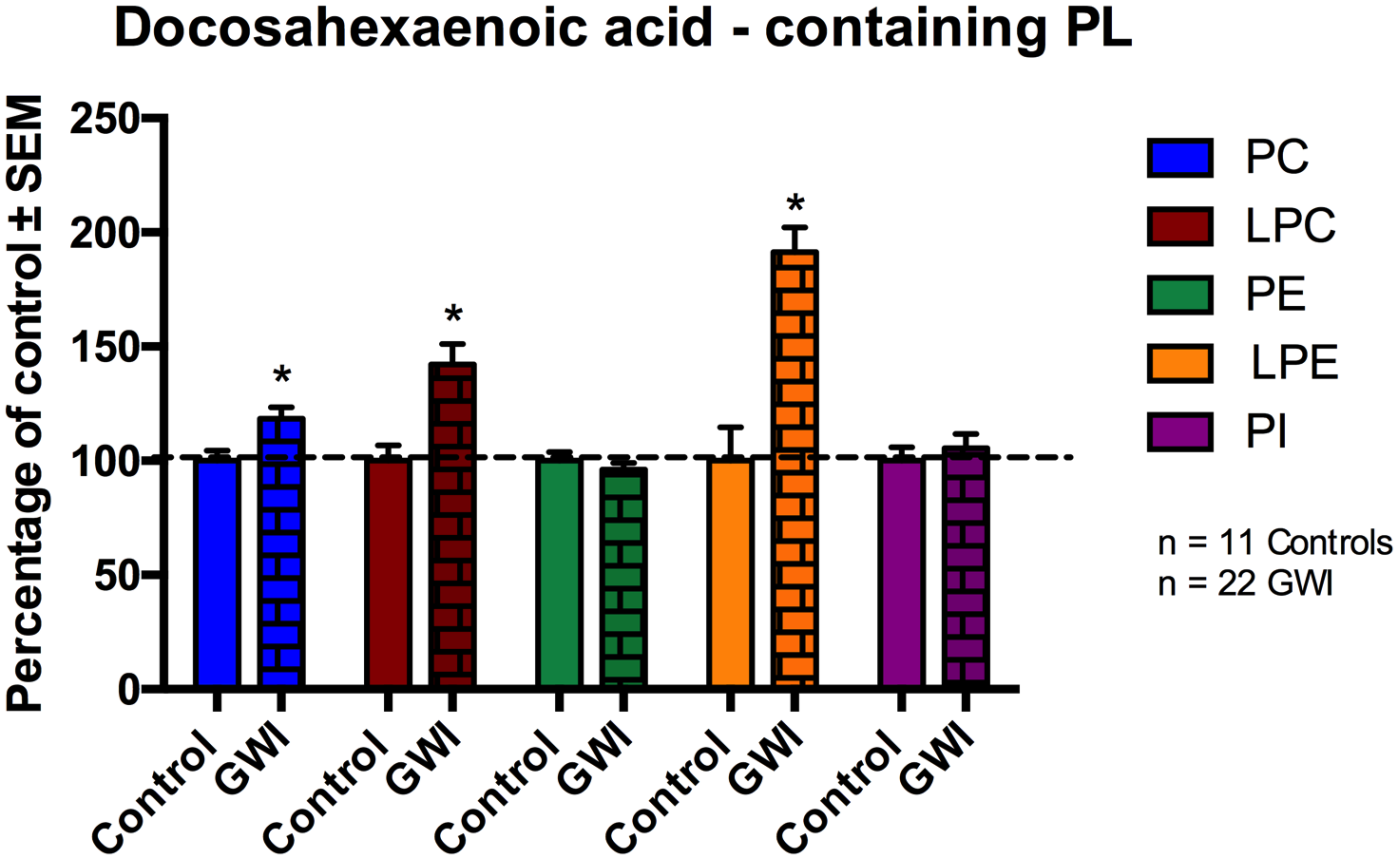
DHA containing phospholipid species in plasma of GWI patients compared to controls, represented in mean ± SEM in percentage of control. Within GWI patients (brick bars), DHA containing PC, LPC and LPE species were significantly elevated compared to controls (solid bars). No differences were observed within DHA containing PE and PI species. *p<0.05; MLM regression with *post hoc* analysis.

Fig 5 (Fig 5) shows individual DHA containing PL species in the plasma of GWI subjects compared to controls. There were no differences between the two groups for DHA containing PE and PI species. However, DHA species of PC were 18% higher in GWI patients compared to controls (p = 0.0013). Furthermore, within LPC and LPE, DHA containing species were increased in GWI compared to controls by 42% (p = 0.001) and 91% (p<0.001), respectively (p values for PE (gender), LPC and PI (age) were adjusted as before).

### Comparison of individual molecular PL species across GWI rodent models and a GW veteran cohort

In order to examine the value of the described rodent models, we investigated individual PL molecular species in these previously published animal models of GWI (rat model: PB+PER+DEET and stress exposure, mouse model: PB+PER exposure) and compared them with the clinical findings. Total PL, degree of saturation, ether and AA and DHA content PL data for both models can be found in S1–S4 Tables. For the comparison of individual molecular species we included PL in our analysis that showed a >1.1 fold change in humans for all PL classes and examined those in the rodent GWI models.

**Fig 6:**
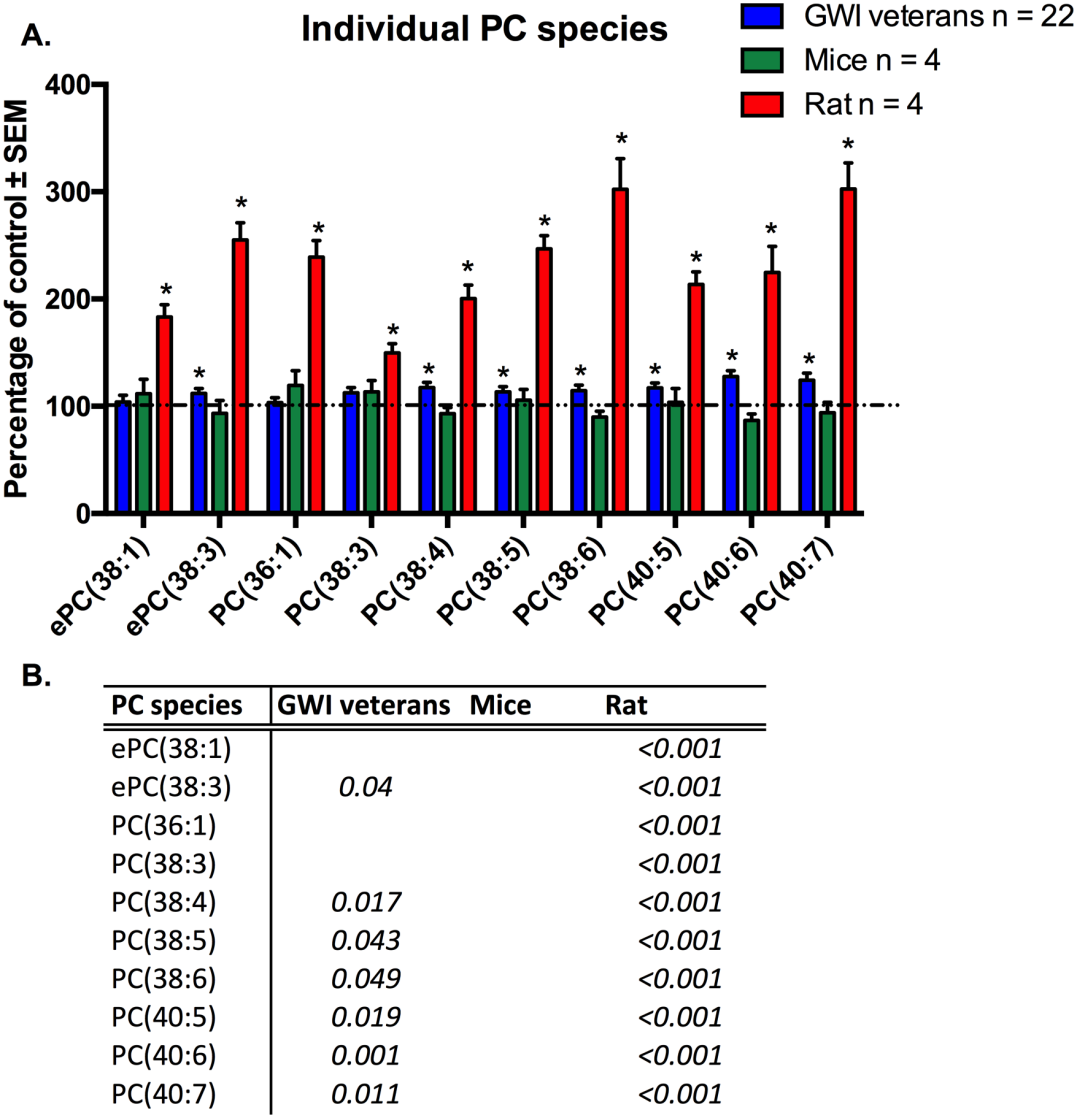
Individual molecular species of PC are elevated in plasma from veterans with GWI, rodent model and mouse model of GWI. A. (blue) Percentage of control ± SEM (n = 11 controls, 22 GWI) showing elevated PC species in GWI subjects compared to controls. (green) Percentage of control ± SEM (n = 4 per group) for the PB+PER model showing that most of these species were also elevated in exposed mice but did not reach statistical significance. (red) Percentage of control ± SEM (n = 6 sham, n = 5 exposed rats) showing elevated PC species in GW agent exposed rats compared to control rats. B. P values for all significant PL species in GW veterans and rodent models. *p<0.05; MLM regression with *post hoc* analysis.

Individual molecular species of PC that were significant for at least one model or the human subject cohort are shown in Fig 6 (Fig 6). Human GWI subjects showed significant increases in 7 out of the 10 shown individual PC species. The highest changes were observed for PC(40:6) and PC(40:7) (p<0.05).Increases ranged from 12% to 28%. Although the remaining 3 species showed increased levels in GWI patients, significance was not achieved. For C57Bl6/J mice PC species ePC(38:1), PC(36:1) and PC(38:3) were non significantly elevated in exposed compared to controls (p>0.05). As with GWI subjects, exposed rats showed significant increases of the above-mentioned PC species, as well as the remaining 3 species compared to controls (p<0.05).

**Fig 7:**
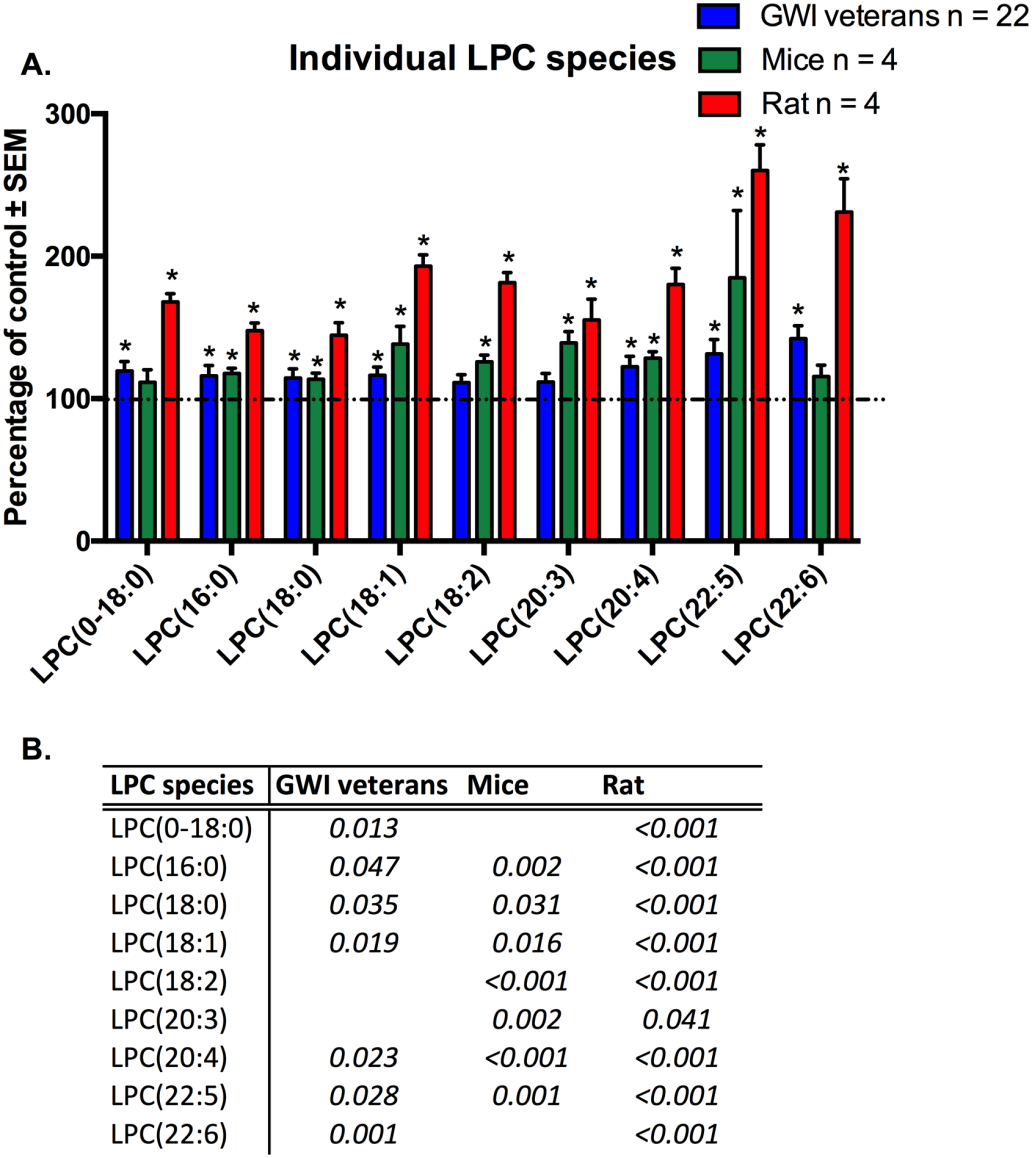
Individual molecular species of LPC are elevated in plasma from veterans with GWI, rodent model and mouse model of GWI. A. (blue) Percentage of control ± SEM (n = 11 controls, 22 GWI) showing elevated LPC species in GWI subjects compared to controls. (green) Percentage of control ± SEM (n = 4 per group) for the PB+PER model showing that most of these species were also elevated in exposed mice. (red) Percentage of control ± SEM (n = 6 sham, n = 5 exposed rats) showing elevated LPC species in GW agent exposed rats compared to control rats. B. P values for all significant PL species in GW veterans and rodent models.*p<0.05; MLM regression with *post hoc* analysis.

Individual molecular species of LPC that were significant for at least one model or the human subject cohort are shown in Fig 7 (Fig 7). After adjusting for age, we observed significantly increased levels for 7 out of the 9 shown molecular lipid species for the GWI patients compared to controls. Levels increased in between the range of 19% (LPC(0–18:0)) to 42% (LPC(22:6n3)). Although the remaining 2 LPC species (LPC(18:2n6) and LPC(20:3)) did not reach significance, a trend (p = 0.06–0.07) for the elevation was observed. For the GWI mouse model, statistical significance was achieved for 6 out of the 9 individual molecular LPC species, including LPC(18:2n6) and LPC(20:4n6) (p<0.05); the remaining 3 showed an a trend increase in the PB+PER mouse group (LPC(22:6n3), p = 0.069, LPC(0–18:0), p = 0.071, LPC(22:5n3), p = 0.063). The rat GWI model showed a significant increase of all shown LPC species in the exposure group (p<0.05), with percent change ranging from 44% (LPC(18:0)) to 160% (LPC(22:5n3)).

**Fig 8:**
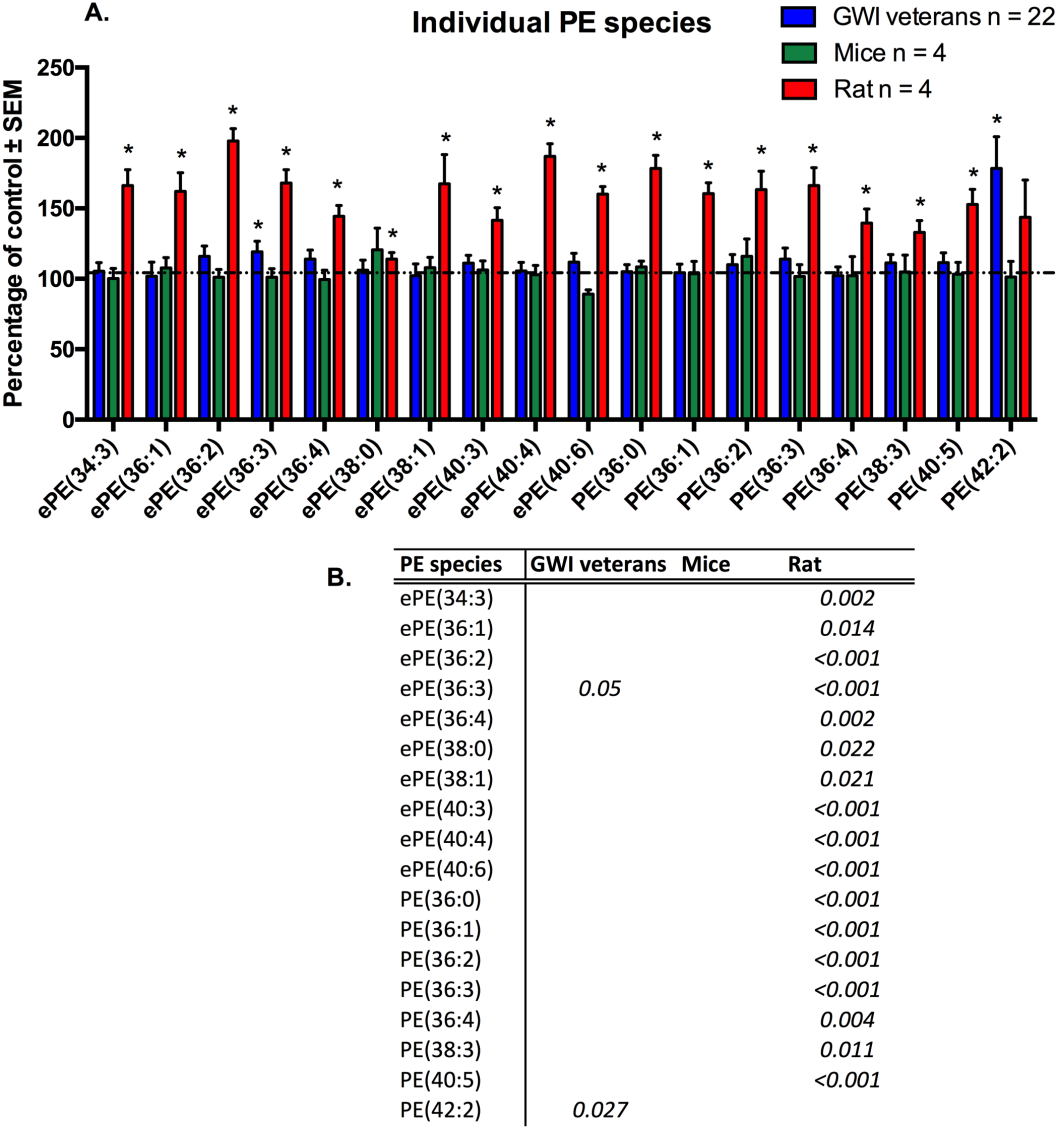
Individual molecular species of PE are elevated in plasma from veterans with GWI, rodent model and mouse model of GWI. A. (blue) Percentage of control ± SEM (n = 11 controls, 22 GWI) showing elevated PE species in GWI subjects compared to controls. (green) Percentage of control ± SEM (n = 4 per group) for the PB+PER model showing that most of these species were also elevated in exposed mice but did not reach statistical significance. (red) Percentage of control ± SEM (n = 6 sham, n = 5 exposed rats) showing elevated LPC species in GW agent exposed rats compared to control rats. B. P values for all significant PL species in GW veterans and rodent models.*p<0.05; MLM regression with *post hoc* analysis.

Individual molecular species of PE that were significant for at least one model or the human subject cohort, are shown in Fig 8 (Fig 8). For human subjects, although we observed tendencies of elevated PE levels in the GWI patient group, only two molecular species reached significance (p<0.05, ePE(36:3) and PE(42:2)). Ether PE(36:3) was elevated by 19% and PE(42:2) by 78%. P values were adjusted for gender effect. Within the mouse model variance was high and no molecular species was significant (p>0.05). PE species ePE(38:0) and PE(36:2) were non-significantly elevated.

Exposed rats showed the strongest signal and significant increases of all shown PE species in the PB+PER+DEET and stress group, except one (p>0.05; PE(42:2).

**Fig 9:**
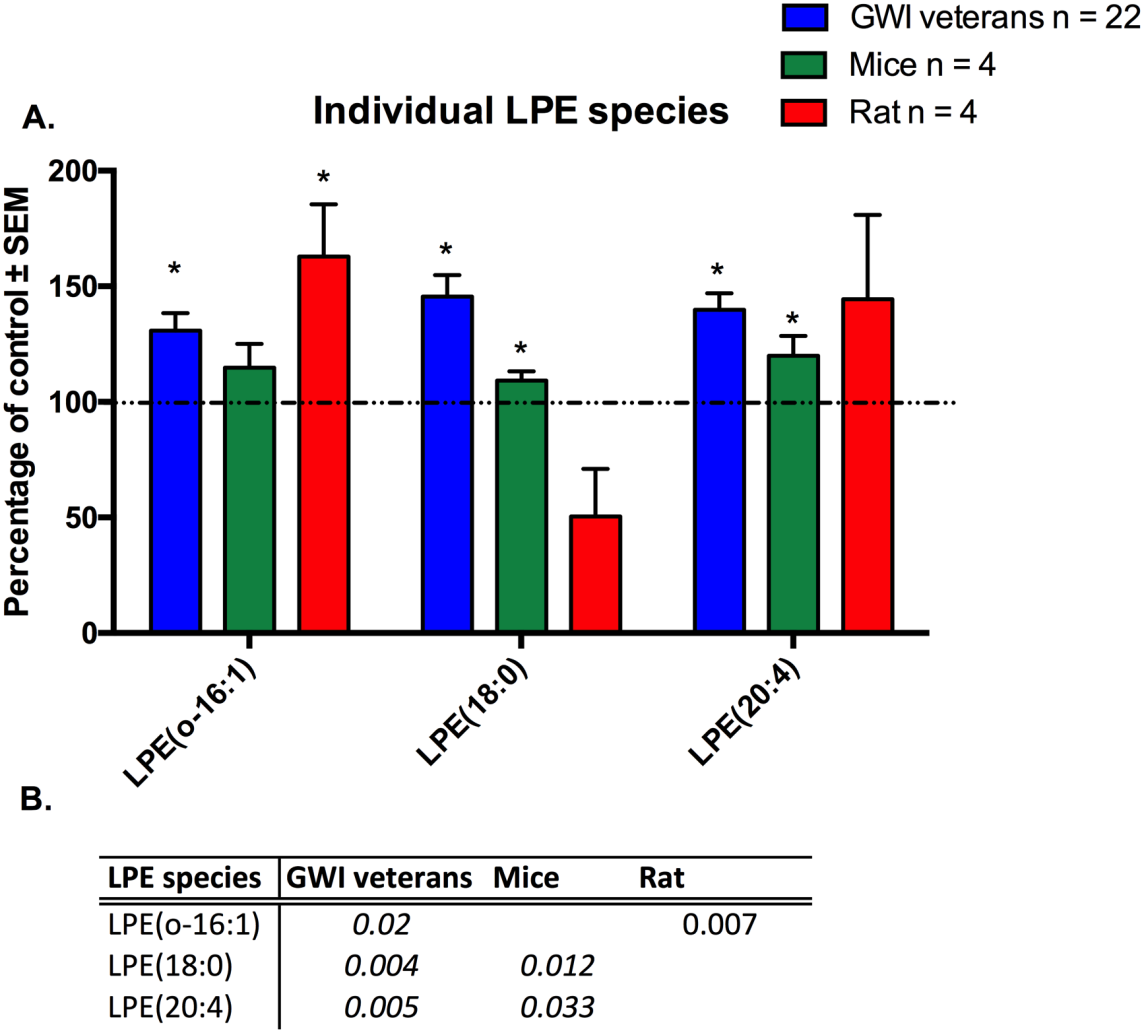
Individual molecular species of LPE are elevated in plasma from veterans with GWI, rodent model and mouse model of GWI. A. (blue) Percentage of control ± SEM (n = 11 controls, 22 GWI) showing elevated LPE species in GWI subjects compared to controls. (green) Percentage of control ± SEM (n = 4 per group) for the PB+PER model showing that two out of three species were also elevated in exposed mice. (c) Percentage of control ± SEM (n = 6 sham, n = 5 exposed rats) showing elevated LPE species in GW agent exposed rats compared to control rats. B. P values for all significant PL species in GW veterans and rodent models. *p<0.05; MLM regression with *post hoc* analysis.

Individual molecular species of LPE that were significant for at least one model or the human subject cohort, are shown in Fig 9 (Fig 9). For LPE, all shown individual molecular species were significantly elevated in human GWI patients compared to control (p<0.05, LPE(0–16:1), LPE(18:0) and LPE(20:4)), with increases between 31% for LPE(0–16:1) and 46% for LPE(18:0). The mouse model showed 2 species, which overlapped in humans that were elevated as well, namely for LPE(18:0) (9%) and LPE(20:4) (20%; p<0.05), whereas the rat model only showed a significant increase for LPE(0–16:1) by 63% (p<0.05).

**Fig 10:**
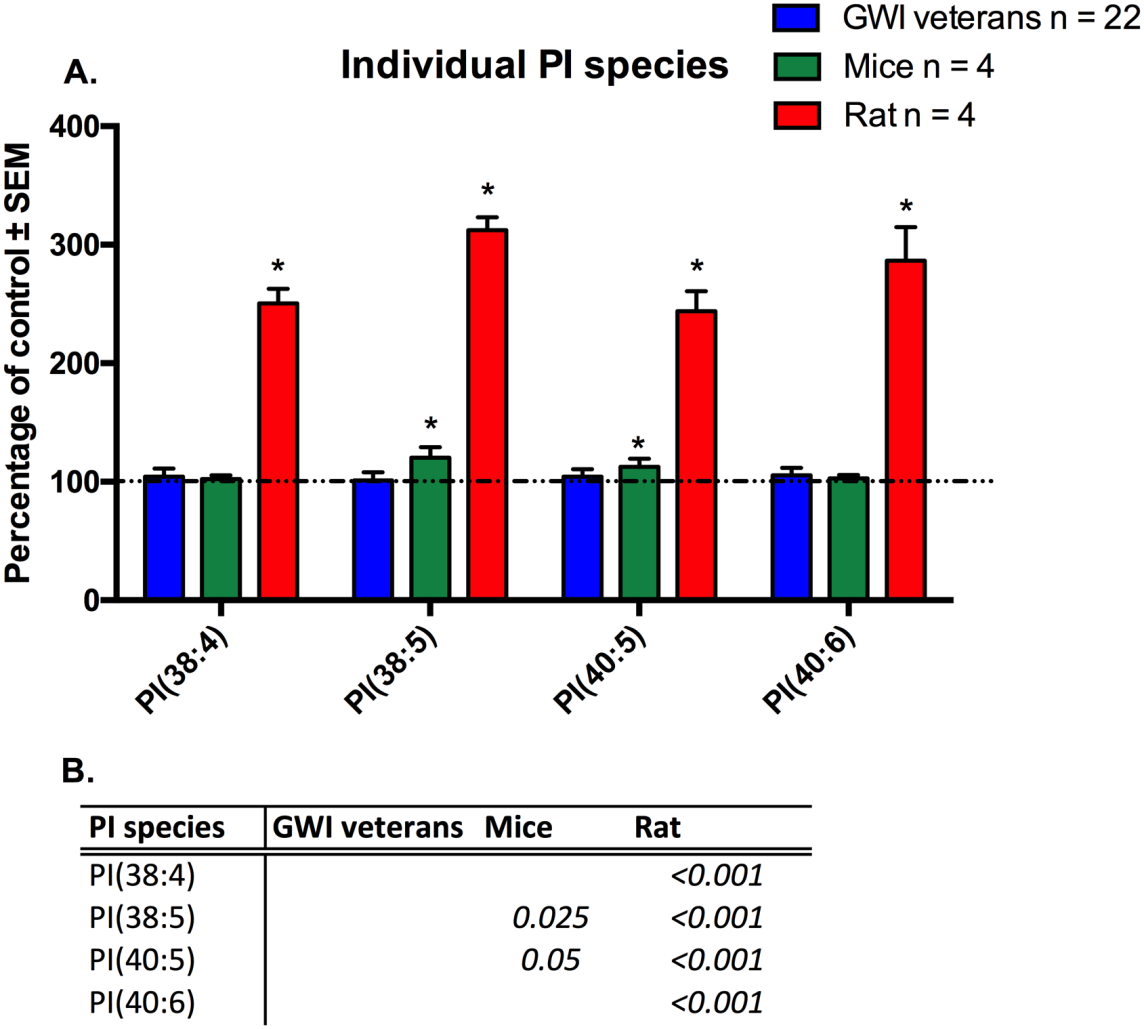
Individual molecular species of PI are elevated in plasma from veterans, rodent model and mouse model of GWI. A. (blue) Percentage of control ± SEM (n = 11 controls, 22 GWI) showing levels PI species in GWI subjects compared to controls. No significance was achieved for any of these species. (green) Percentage of control ± SEM (n = 4 per group) for the PB+PER model showing that 2 out of 5 species were elevated in exposed mice. (red) Percentage of control ± SEM (n = 6 sham, n = 5 exposed rats) showing elevated PI species in GW agent exposed rats compared to control rats. B. P values for all significant PL species in GW veterans and rodent models.*p<0.05; MLM regression with *post hoc* analysis.

Individual molecular species of PI that were significant for at least one model or the human subject cohort, are shown in Fig 10 (Fig 10). For PI, none of the shown individual molecular species were significantly different in human GWI patients compared to control (p>0.05). P value was adjusted for age effect. PB+PER mice showed increased levels for two out of the five PI species, PI(38:5) and PI(40:5) (p<0.05) by 20% and 13% respectively. In correlation, exposed rats showed in correlation a significant increase for all PI species ((PI38:4), PI(38:5), PI(40:5) and PI (40:6), with elevations ranging from 143% to 212%.

## Discussion

Shortly after returning from the 1991 conflict, a large proportion of GW veterans presented multiple symptoms, later defined as GWI. The heterogeneous clinical presentation has made it difficult to objectively diagnose GWI, identify underlying pathology and find effective treatments for this debilitating illness. Since animal modeling allows for better control of genetic heterogeneity and environment factors, evaluation of GW exposures in rodents therefore allow us to identify outcomes that are directly related to GW chemical exposure. As such, identification of overlapping changes in GWI rodent models and human patients will likely facilitate development of diagnostic and therapeutic approaches that are relevant to the underlying pathological features of GWI. In this study, we performed PL profiling to identify areas of overlap between the clinical patient population and GWI animal models.

As reported above, the PB+PER mouse model and the PB+PER+DEET and stress rat model utilized in this study have been well characterized with respect to neurobehavioral features that are similar to chronic symptoms reported by GWI patients [9,10,41,44,47]. Samples from both rodent models were collected at 6 months post exposure. This six month waiting period after exposure of rodents to GW agents is equivalent to about 17 years after exposure in humans [63]. This waiting period is relevant as >25 years have passed since soldiers were exposed to these chemicals during the GW. Therefore, these experiments evaluate an extended (chronic) time point after exposure to GW agents, which is relevant to the current clinical condition of veterans with GWI. As such, these rodent models are ideal candidates for translational studies aimed at examining long-term changes that are relevant to chronic features of GWI symptomatology.

Evaluation of plasma PL revealed elevated levels of total LPC in veterans with GWI compared to controls. Both mouse and rat models also showed significantly higher total LPC levels in exposed animals compared to controls. For total PC, PE and PI there were no significant differences between the groups for humans and mouse studies. However, exposed rats showed a strong increase in these PL classes. Because rats were exposed to stress and DEET, in addition to PB+PER, future investigation in subtypes of exposure in relation to GWI severity and symptom profiles may provide insights into possible differential response to GW agents and PL profiles.

We also examined the degree of unsaturation of different PL classes and found a good overlap for LPC, with all SFA, MUFA and PUFA being elevated in GWI patients and in exposed rodents in the GWI animal models (see S1 Table). In particular, PUFA LPC species reached statistical significance in both rodent GWI models and in human GWI patients. Yet, differences between rodents and humans for example were found for PE. For example, no significance was observed in humans, however mice showed lower SFA and PUFA containing PE species, whereas rats had increased levels of saturated and unsaturated PE. These studies suggest possible differences in PL profiles in response to different combinations of GW exposures and chronicity of illness, which warrant further investigation. As most of these PL species in plasma are bound to various lipoproteins, differences between human and rodent lipoprotein compositions could influence these findings [64]. For example, HDL particles, which contain PL, differ in humans and rodents in regard to their size patterns [65], which could affect the profiles of certain PL classes between human and rodents. Therefore, it may be helpful to further study the lipoprotein composition in rodents in order to understand the complexity of our findings. Furthermore, it is known that SFA and MUFA can be synthesized *de novo* within the brain, yet essential PUFA are mainly supplied from the periphery [61]. Plasma non-esterified-DHA is the major pool, which supplies the brain with DHA[66]. Additionally, LPC (22:6n3) can be directly transported from plasma to the brain via the specialized receptors mfsd2a receptor [67]. As such, increases in essential PUFA containing PL species could indicate transport deficiencies into various organs, including the brain. However, further investigation of PL content in the brain is necessary to fully understand the reasons behind those changes.

Additionally, shorter chain PUFA can be used to synthesize their longer chain fatty acid counterparts, such as AA (20:4n6) and DHA (22:6n3), which are involved in regulating the intensity and duration of inflammatory responses [68]; possible changes in their levels could influence the immune/inflammatory imbalances observed in GWI [24]. Our findings in relation to changes in PUFA, particularly AA and DHA containing PL (e.g. increased DHA containing PC species in humans and rats), suggest that the examination of bioactive metabolites of AA and DHA in relation to GWI pathology in the future could be useful. Interestingly DHA and AA containing PC species were also increased in the brains of 5 month old PB+PER GWI mice [34]. However, further studies investigating lipid enzymes, transport and metabolism are necessary to understand the relationship between brain and peripheral changes. Yet, this investigation and previous studies clearly underline the importance of lipid modulation in GWI pathology [16,19,34,52].

We observed changes in ether containing PL species. As ether PL are dependent upon peroxisomes for their synthesis, examination of their functionality should be investigated in the future. Within all PL classes, only eLPE showed elevation in GWI patients compared to control. However, in mice no ether lipid class was significantly changed, whereas rats showed significant increases for ePC, eLPC and ePE. Again, one possibility could be that the elevated stress dose in rats leads to changes in ether lipids, which are not observed in humans or mice.

We compared individual molecular species of each PL class within rats and mice to human plasma samples to identify species with diagnostic potential and to investigate overlap between the GWI models and veterans with this condition with the goal of facilitating future translational work aimed at developing therapies for treating GWI. We observed increases in all classes for overlapping molecular species. The most consistency was seen for several individual LPC species, such as LPC(16:0), LPC(18:0), LPC(18:1n9) and LPC(20:4n6).

The rat model showed the strongest signal with elevations reaching up to 200% for all PL classes. Some epidemiological studies in GW veterans have shown that operational stress amplifies the effects of GW agents, leading to more severe symptom presentations of GWI [6,42,43]. These findings have been supported by exercise challenges in humans showing exercise induced hyperalgesia in a GWI subpopulation as well as alterations in immune functioning, cognition and brain structure [69]. Moreover animal studies have shown, that combined GW agent and stress exposure induce long-term behavioral consequences as well as long-term learning dysfunction in a rat model of GWI [19,20]. Because of this it would be interesting to include a stress paradigm for veterans and collect plasma samples of GWI veterans after exercise challenge to determine if lipid metabolism is further disturbed.

The most significant changes were observed for LPC and LPE, within the GWI patients compared to controls. Since these PL can be generated by phospholipase action on PC and PE [70], additional investigation is needed to explain the observed changes.

Our human sample size was small and therefore we were not able to fully assess the impact of gender and age on PL levels in this case control study, but instead we adjusted our statistical analyses to account for these confounding factors. Further validation is required in a larger cohort to accomplish this. Validation studies using a larger cohort of GWI and control GW veterans will also allow us the opportunity to assess differential response to various combinations of exposure to GW agents. We further will be able to investigate differences in lipid profiles based on the severity of symptoms and different symptom clusters in order to identify subgroups of veterans with GWI. This could ultimately improve our ability to provide an objective diagnosis and management of relevant symptoms in subgroups of ill veterans. Use of i.p. in our mouse model for PB and PER mouse model is also a limitation [71–73] but this method helps with reducing the variability associated with chemical delivery to mice [71,72]. Furthermore, the observed outcomes are similar to those reported in other rodent models of GWI at the chronic post-exposure time points, which use oral and the topical routes [44]. As such, the chronic effects of GW agent exposure appear to be unaffected by the mode of delivery [44–46,74].

## Conclusion

In this study, we identified significantly different overlapping phospholipid changes between two different rodent models of GWI and a clinical sample of veterans with GWI. These differences were particularly evident within the lysophospholipids. This first attempt at a pilot translational study using two different rodent models and a clinical cohort of veterans with GWI proved successful in suggesting strong potential avenues for further larger studies of phospholipid biomarkers of GWI. These results also provide evidence for further study of these models for therapeutic target discovery if validated in larger preclinical and clinical samples.

## Supporting information

S1 Table Principal component analysis for human samples showing factors and total variance associated with each component, which were significantly associated with exposure based on MLM analyses. In humans, for PC 8 components were identified with PCA analysis, of which components 2 (p = 0.005), 4 (p<0.001), 5 (p = 0.006) and 6 (p = 0.032) were significant for GWI diagnosis. For LPC 8 components were identified of which component 3 was significant for GWI (p = 0.003). For PE, component 4 was significant (p = 0.014) out of 8 identified. For LPE, out of 4 identified components, component 1 was significant (p<0.001) for GWI. For PI only one component was identified and significant via PCA (p = 0.002). For SM, 3 components were identified with PCA analysis, of which component 1 was significant for GWI (p = 0.032).
(DOCX)

S2 Table Principal component analysis for mice samples showing factors and total variance associated with each component, which were significantly associated with exposure based on MLM analyses. In mice, for PC 5 components were identified with PCA analysis, of which component 4 (p = 0.005) was significant for PB+PER exposure. For LPC 4 components were identified of which component 1 was significant for PB+PER exposure (p<0.001). For PE, component 1 was significant (p = 0.036) out of 5 identified. For LPE, out of 3 identified components, component 1 was significant (p = 0.009) for GW agent exposure. For PI 4 components were identified and significant via PCA for component 3 (p = 0.008). For SM, 3 components were identified with PCA analysis, of which component 3 was significant for PB+PER (p = 0.044).
(DOCX)

S3 Table Principal component analysis for rat samples showing factors and total variance associated with each component, which were significantly associated with exposure based on MLM analyses. In rats, for PC 3 components were identified with PCA analysis, of which component 1 (p = 0.017) and 2 (p<0.001) were significant for PB+PER+DEET and stress exposure. For LPC only 1 component was identified and significant for PB+PER+DEET and stress exposure (p<0.001). For PE, components 2 (p = 0.003), 4 (p = 0.017), and 5 (p = 0.001) were significant out of 5 identified. For LPE, out of 3 identified components, component 2 was significant (p = 0.003) for GW agent and stress exposure. For PI 3 components were identified and significant via PCA for component 1 (p<0.001). For SM, 1 component was identified with PCA analysis and significant for PB+PER+DEET and stress exposure (p<0.001).
(DOCX)

S4 Table Total plasma levels for human samples, mice and rat model of PC, LPC, PE, LPE, PI and SM quantified by LC/MS analyses (all values are presented in μM ± SEM). Individual molecular species of each class were quantified by LC/MS and summed after lipidomeDB analyses to generate total lipid levels. *denotes significant p values for p<0.05.
(DOCX)

S5 Table Plasma levels for humans, mice and rat model of SFA, MUFA and PUFA containing PC, LPC, PE, LPE and PI species quantified by LC/MS analyses (all values are presented in μM ± SEM). *denotes significant p values for p<0.05.
(DOCX)

S6 Table Plasma levels for humans, mice and rat model of ether containing PC, LPC, PE and LPE species quantified by LC/MS analyses (all values are presented in μM ± SEM). *denotes significant p values for p<0.05.
(DOCX)

S7 Table Plasma levels for humans, mice and rat model of AA and DHA containing PC, LPC, PE and LPE species quantified by LC/MS analyses (all values are presented in μM ± SEM). No DHA containing LPE species were identified for the rat model, therefore ratio for AAtoDHA containing LPE species could not be determined.*denotes significant p values for p<0.05.
(DOCX)

## References

[1] Binns J, Barlow C, Bloom F, Clauw D, Golomb B, Graves J, et al Gulf War Illness and the Health of Gulf War Veterans: Scientific Findings and Recommendations. 2008; 1–465.

[2] Fukuda K, Nisenbaum R, Stewart G, Thompson WW, Robin L, Washko RM, et al Chronic multisymptom illness affecting Air Force veterans of the Gulf War. JAMA. 1998;280: 981–988. 9749480 10.1001/jama.280.11.981

[3] von Bohlen und Halbach O. Analysis of morphological changes as a key method in studying psychiatric animal models. Cell Tissue Res. 2013;354: 41–50. 10.1007/s00441-012-1547-9 23334194 PMC3785701

[4] Fulco C, Liverman C, Sox H. Gulf War and Health: Volume 2 Insecticides and Solvents.; Fulco CE, Liverman CT, Sox HC, editors: N.R.C. The National Academies Press; 2003. 25057724

[5] Steele L. Prevalence and patterns of Gulf War illness in Kansas veterans: association of symptoms with characteristics of person, place, and time of military service. Am J Epidemiol. 2000;152: 992–1002. 11092441 10.1093/aje/152.10.992

[6] Unwin C, Blatchley N, Coker W, Ferry S, Hotopf M, Hull L, et al Health of UK servicemen who served in Persian Gulf War. Lancet (London, England). 1999;353: 169–178. 10.1016/S0140-6736(98)11338-7 9923871

[7] White RF, Steele L, O’Callaghan JP, Sullivan K, Binns JH, Golomb BA, et al Recent research on Gulf War illness and other health problems in veterans of the 1991 Gulf War: Effects of toxicant exposures during deployment. Cortex. Elsevier Ltd; 2015;1. 10.1016/j.cortex.2015.08.022 PMC4724528 26493934

[8] RAC. Gulf War Illness and the Health of Gulf War Veterans: Research Update and Recommendations, 2009–2013 Updated Scientific Findings and Recommendations. 2014; 1–123.

[9] Toomey R, Alpern R, Vasterling JJ, Baker DG, Reda DJ, Lyons MJ, et al Neuropsychological functioning of U.S. Gulf War veterans 10 years after the war. J Int Neuropsychol Soc JINS. 2009;15: 717–729. 10.1017/S1355617709990294 19640317

[10] Sullivan K, Krengel M, Proctor SP, Devine S, Heeren T, White RF. Cognitive Functioning in Treatment-Seeking Gulf War Veterans: Pyridostigmine Bromide Use and PTSD. J Psychopathol Behav Assess. 2003;25: 95–103.

[11] David AS, Farrin L, Hull L, Unwin C, Wessely S, Wykes T. Cognitive functioning and disturbances of mood in UK veterans of the Persian Gulf War: a comparative study. Psychol Med. 2002;32: 1357–1370. 12455934 10.1017/s0033291702006359

[12] Vythilingam M, Luckenbaugh DA, Lam T, Morgan CA, Lipschitz D, Charney DS, et al Smaller head of the hippocampus in Gulf War-related posttraumatic stress disorder. Psychiatry Res. 2005;139: 89–99. 10.1016/j.pscychresns.2005.04.003 15967648

[13] Smith BN, Wang JM, Vogt D, Vickers K, King DW, King LA. Gulf War Illness: Symptomatology Among Veterans 10 Years After Deployment Brian. JOEM. 2013;55. 10.1097/JOM.0b013e318270d709 23235463

[14] Abdel-Rahman A, Abou-Donia SM, El-Masry EM, Shetty AK, Abou-Donia MB. Stress and Combined Exposure to Low Doses of Pyridostigmine Bromide, DEET, and Permethrin Produce Neurochemical and Neuropathological Alterations in Cerebral Cortex, Hippocampus, and Cerebellum. J Toxicol Environ Heal Part A. 2004;67: 163–192. 10.1080/15287390490264802 14675905

[15] Abou-Donia MB, Dechkovskaia AM, Goldstein LB, Abdel-Rahman A, Bullman SL, Khan WA. Co-exposure to pyridostigmine bromide, DEET, and/or permethrin causes sensorimotor deficit and alterations in brain acetylcholinesterase activity. Pharmacol Biochem Behav. 2004;77: 253–262. 14751452 10.1016/j.pbb.2003.10.018

[16] Abdullah L, Crynen G, Reed J, Bishop A, Phillips J, Ferguson S, et al Proteomic CNS profile of delayed cognitive impairment in mice exposed to Gulf War agents. Neuromolecular Med. 2011;13: 275–88. 10.1007/s12017-011-8160-z 21986894

[17] White RF, Proctor ÃSP, Heeren T, Wolfe J, Krengel M, Vasterling J, et al Neuropsychological Function in Gulf War Veterans: Relationships to Self-Reported Toxicant Exposures. 2001;54: 42–54. 10.1002/ajim.1070 11439396

[18] White RF. Recent research on Gulf War illness and other health problems in veterans of the 1991 Gulf War: Effects of toxicant exposures during deployment. Cortex. 2016;74: 449–475. 10.1016/j.cortex.2015.08.022 26493934 PMC4724528

[19] Lamproglou I, Barbier L, Diserbo M, Fauvelle F, Fauquette W, Amourette C. Repeated stress in combination with pyridostigmine. Part I: Long-term behavioural consequences. Behav Brain Res. 2009;197: 301–310. 10.1016/j.bbr.2008.08.031 18793677

[20] Barbier L, Diserbo M, Lamproglou I, Amourette C, Peinnequin A, Fauquette W. Repeated stress in combination with pyridostigmine Part II: changes in cerebral gene expression. Behav Brain Res. 2009;197: 292–300. 10.1016/j.bbr.2008.08.032 18796314

[21] Amourette C, Lamproglou I, Barbier L, Fauquette W, Zoppe A, Viret R, et al Gulf War illness: Effects of repeated stress and pyridostigmine treatment on blood-brain barrier permeability and cholinesterase activity in rat brain. Behav Brain Res. 2009;203: 207–214. 10.1016/j.bbr.2009.05.002 19433115

[22] Terry AV Jr. Functional Consequences of Repeated Organophosphate Exposure: Potential Non-Cholinergic Mechanisms. Pharmacol Ther. 2012;134: 335–365. 10.1016/j.pharmthera.2012.03.001 PMC3366364 22465060

[23] Phillips KF, Deshpande LS. Repeated low-dose organophosphate DFP exposure leads to the development of depression and cognitive impairment in a rat model of Gulf War Illness. Neurotoxicology. Elsevier B.V.; 2016;52: 127–133. 10.1016/j.neuro.2015.11.014 26619911

[24] O’Callaghan JP, Kelly K a, Locker AR, Miller DB, Lasley SM. Corticosterone primes the neuroinflammatory response to DFP in mice: potential animal model of Gulf War Illness. J Neurochem. 2015; 1–14. 10.1111/jnc.13088 PMC4722811 25753028

[25] Skowera A, Hotopf M, Sawicka E, Varela-Calvino R, Unwin C, Nikolaou V, et al Cellular immune activation in Gulf War veterans. J Clin Immunol. 2004;24: 66–73. 10.1023/B:JOCI.0000018065.64685.82 14997036

[26] Broderick G, Kreitz A, Fuite J, Fletcher MA, Vernon SD, Klimas N. A pilot study of immune network remodeling under challenge in Gulf War Illness. Brain Behav Immun. 2011;25: 302–313. 10.1016/j.bbi.2010.10.011 20955779

[27] Broderick G, Ben-Hamo R, Vashishtha S, Efroni S, Nathanson L, Barnes Z, et al Altered immune pathway activity under exercise challenge in Gulf War Illness: An exploratory analysis. Brain Behav Immun. Elsevier Inc.; 2013;28: 159–169. 10.1016/j.bbi.2012.11.007 23201588

[28] Smylie AL, Broderick G, Fernandes H, Razdan S, Barnes Z, Collado F, et al A comparison of sex-specific immune signatures in Gulf War illness and chronic fatigue syndrome. BMC Immunol. BMC Immunology; 2013;14: 29 10.1186/1471-2172-14-29 23800166 PMC3698072

[29] Whistler T, Fletcher MA, Lonergan W, Zeng X-R, Lin J-M, Laperriere A, et al Impaired immune function in Gulf War Illness. BMC Med Genomics. 2009;2: 12 10.1186/1755-8794-2-12 19265525 PMC2657162

[30] Khaiboullina SF, DeMeirleir KL, Rawat S, Berk GS, Gaynor-Berk RS, Mijatovic T, et al Cytokine expression provides clues to the pathophysiology of Gulf War illness and myalgic encephalomyelitis. Cytokine. Elsevier Ltd; 2015;72: 1–8. 10.1016/j.cyto.2014.11.019 PMC4410698 25514671

[31] Farooqui AA, Harroocks LA, Farooqui T. Interactions Between Neural Membrane Glycerophospholipid and Sphingolipid Mediators: A Recipe for Neural Cell Survival or Suicide. J Neurosci Res. 2007;85: 1834–1850. 10.1002/jnr.21268 17393491

[32] Serhan CN, Yacoubian S, Yang R. Anti-inflammatory and proresolving lipid mediators. Annu Rev Pathol. 2008;3: 279–312. 10.1146/annurev.pathmechdis.3.121806.151409 18233953 PMC2739396

[33] Fredman G, Serhan CN. Specialized proresolving mediator targets for RvE1 and RvD1 in peripheral blood and mechanisms of resolution. Biochem J. 2011;437: 185–197. 10.1042/BJ20110327 21711247 PMC3133883

[34] Abdullah L, Evans JE, Montague H, Reed JM, Moser A, Crynen G, et al Chronic elevation of phosphocholine containing lipids in mice exposed to Gulf War agents pyridostigmine bromide and permethrin. Neurotoxicology Teratol. 2013;40: 74–84. 10.1016/j.ntt.2013.10.002 24140745

[35] Amenta F, Tayebati SK. Pathways of acetylcholine synthesis, transport and release as targets for treatment of adult-onset cognitive dysfunction. Curr Med Chem. 2008;15: 488–498. 18289004 10.2174/092986708783503203

[36] Koslik HJ, Hamilton G, Golomb BA. Mitochondrial dysfunction in Gulf War illness revealed by 31phosphorus magnetic resonance spectroscopy: A case-control study. PLoS One. 2014;9: 1–6. 10.1371/journal.pone.0092887 PMC3968048 24675771

[37] Golomb BA. Oxidative Stress and Mitochondrial Injury in Chronic Multisymptom Conditions: From Gulf War Illness to Autism Spectrum Disorder. Nat Preceedings. 2012;

[38] Golomb B, Allison M, Koperski S, Koslik H. Coenzyme Q10 Benefits Symptoms in Gulf War Veterans: Results of a Randomized Double-Blind Study. Neural Comput. 2014;1872: 1840–1872. 10.1162/NECO_a_00659 25149705

[39] Mejia EM, Hatch GM. Mitochondrial phospholipids: role in mitochondrial function. J Bioenerg Biomembr. Journal of Bioenergetics and Biomembranes; 2016;48: 99–112. 10.1007/s10863-015-9601-4 25627476

[40] Murphy EJ, Owada Y, Kitanaka N, Kondo H, Glatz JFC. Brain arachidonic acid incorporation is decreased in heart fatty acid binding protein gene-ablated mice. Biochemistry. 2005;44: 6350–6360. 10.1021/bi047292r 15835924

[41] Zakirova Z, Tweed M, Crynen G, Reed J, Abdullah L, Nissanka N, et al Gulf War agent exposure causes impairment of long-term memory formation and neuropathological changes in a mouse model of Gulf War Illness. PLoS One. 2015;10: e0119579 10.1371/journal.pone.0119579 25785457 PMC4364893

[42] Gray GC, Kaiser KS, Hawksworth AW, Hall FW, Barrett-Connor E. Increased postwar symptoms and psychological morbidity among U.S. Navy Gulf War veterans. Am J Trop Med Hyg. 1999;60: 758–766. 10344649 10.4269/ajtmh.1999.60.758

[43] Spencer PS, McCauley LA, Lapidus JA, Lasarev M, Joos SK, Storzbach D. Self-reported exposures and their association with unexplained illness in a population-based case-control study of Gulf War veterans. J Occup Environ Med. 2001;43: 1041–56. 11765675 10.1097/00043764-200112000-00006

[44] Parihar VK, Hattiangady B, Shuai B, Shetty AK. Mood and memory deficits in a model of Gulf War illness are linked with reduced neurogenesis, partial neuron loss, and mild inflammation in the hippocampus. Neuropsychopharmacology. 2013;38: 2348–62. 10.1038/npp.2013.158 23807240 PMC3799073

[45] Hattiangady B, Mishra V, Kodali M, Shuai B, Rao X, Shetty AK. Object location and object recognition memory impairments, motivation deficits and depression in a model of Gulf War illness. Front Behav Neurosci. 2014;8: 78 10.3389/fnbeh.2014.00078 24659961 PMC3952084

[46] Megahed T, Hattiangady B, Shuai B, Shetty AK. Parvalbumin and neuropeptide Y expressing hippocampal GABA-ergic inhibitory interneuron numbers decline in a model of Gulf War illness. Front Cell Neurosci. 2014;8: 447 10.3389/fncel.2014.00447 25620912 PMC4288040

[47] Zakirova Z, Crynen G, Hassan S, Abdullah L, Horne L, Mathura V, et al A Chronic Longitudinal Characterization of Neurobehavioral and Neuropathological Cognitive Impairment in a Mouse Model of Gulf War Agent Exposure. Front Integr Neurosci. 2016;9: 1–24. 10.3389/fnint.2015.00071 PMC4709860 26793076

[48] Hilborne L. Examining possible causes of Gulf War Illness: RAND policy investigations and reviews of the scientific literature RAND Corporation. Santa Monica, CA; 2005 http://www.rand.org/pubs/research_briefs/RB7544

[49] Tanielian T, Jaycox LH, Adamson DM, Burnam MA, Burns RM, Caldarone LB, et al. Invisible Wounds of War [Internet]. 2008.

[50] Dodd CA, Klein BG. Pyrethroid and organophosphate insecticide exposure in the 1-methyl-4-phenyl-1,2,3,6-tetrahydropyridine mouse model of Parkinson’s disease: an immunohistochemical analysis of tyrosine hydroxylase and glial fibrillary acidic protein in dorsolateral striatu. Toxicol Ind Health. 2009;25: 25–31. 10.1177/0748233709102752 19318502

[51] Pittman JT, Dodd CA, Klein BG. Immunohistochemical Changes in the Mouse Striatum Induced by the Pyrethroid Insecticide Permethrin. Int J Toxicol. 2003;22: 359–370. 10.1177/109158180302200504 14555407

[52] Abdullah L, Evans JE, Bishop A, Reed JM, Crynen G, Phillips J, et al Lipidomic profiling of phosphocholine-containing brain lipids in mice with sensorimotor deficits and anxiety-like features after exposure to Gulf War agents. Neuromolecular Med. 2012;14: 349–61. 10.1007/s12017-012-8192-z 22798222

[53] Abdullah L, Evans JE, Joshi U, Crynen G, Reed J, Mouzon B, et al Translational potential of long-term decreases in mitochondrial lipids in a mouse model of Gulf War Illness. Toxicology. Elsevier Ireland Ltd; 2016;372: 22–33. 10.1016/j.tox.2016.10.012 27931520

[54] Chaney LA, Rockhold RW, Hume AS. Cardiorespiratory effects following acute exposure to pyridostigmine bromide and/or N,N-diethyl-m-toluamide (DEET) in rats. Int J Toxicol. 2002;21: 287–300. 10.1080/10915810290096450 12171630

[55] Williamson EG, Long SF, Kallman MJ, Wilson MC. A comparative analysis of the acute toxicity of technical-grade pyrethroid insecticides and their commercial formulations. Ecotoxicol Environ Saf. 1989;18: 27–34. 2776687 10.1016/0147-6513(89)90089-4

[56] Persian Gulf Veterans Coordinating Board. Unexplained Illnesses Among Desert Storm Veterans. Arch Intern Med. 1995; 262–268. 7832597 10.1001/archinte.155.3.262

[57] FOLCH J, LEES M, SLOANE STANLEY GH. A simple method for the isolation and purification of total lipides from animal tissues. J Biol Chem. 1957;226: 497–509. 13428781

[58] Emmerich T, Abdullah L, Crynen G, Dretsch M, Evans J, Ait-Ghenzala G, et al Plasma lipidomic profiling in a military population of mTBI and PTSD with APOE ε4 dependent effect. J Neurotrauma. 2015;33: 1331–48. 10.1089/neu.2015.4061 26714394

[59] Emmerich T, Abdullah L, Ojo J, Mouzon B, Nguyen T, Laco GS, et al Mild TBI Results in a Long-Term Decrease in Circulating Phospholipids in a Mouse Model of Injury. NeuroMolecular Med. Springer US; 2016; 10.1007/s12017-016-8436-4 27540748

[60] Abdullah L, Evans JE, Ferguson S, Mouzon B, Montague H, Reed J, et al Lipidomic analyses identify injury-specific phospholipid changes 3 mo after traumatic brain injury. FASEB J. 2014;28: 5311–5321. 10.1096/fj.14-258228 25208845

[61] Bazinet RP, Layé S. Polyunsaturated fatty acids and their metabolites in brain function and disease. Nat Rev Neurosci. 2014;15: 771–785. 10.1038/nrn3820 25387473

[62] van den Bosch H, Schutgens RBH, Wanders RJA, Tager JM. Biochemistry of peroxisomes. Annu Rev Biochem. 1992;61: 157–97. 10.1146/annurev.bi.61.070192.001105 1353950

[63] Sengupta P. The Laboratory Rat: Relating Its Age With Human’s. Int J Prev Med. 2013;4: 624–630. 23930179 PMC3733029

[64] Gordon S., Li H, Zhu X, Shah A., Lu L., Davidson WS. A Comparison of the Mouse and Human Lipoproteome: Suitability of the Mouse Model for Studies of Human Lipoproteins. J Proteome Res. 2015;14: 2686–2695. 10.1021/acs.jproteome.5b00213 25894274 PMC4712022

[65] Chajek-Shaul T, Hayek T, Walsh A, Breslow JL. Expression of the human apolipoprotein A-I gene in transgenic mice alters high density lipoprotein (HDL) particle size distribution and diminishes selective uptake of HDL cholesteryl esters. Proc Natl Acad Sci U S A. 1991;88: 6731–5. 1907375 10.1073/pnas.88.15.6731 PMC52162

[66] Chen CT, Kitson AP, Hopperton KE, Domenichiello AF, Trépanier M-O, Lin LE, et al Plasma non-esterified docosahexaenoic acid is the major pool supplying the brain. Sci Rep. Nature Publishing Group; 2015;5: 15791. 10.1038/srep15791 PMC4625162 26511533

[67] Nguyen LN, Ma D, Shui G, Wong P, Cazenave-Gassiot A, Zhang X, et al Mfsd2a is a transporter for the essential omega-3 fatty acid docosahexaenoic acid. Nature. Nature Publishing Group; 2014;509: 503–506. 10.1038/nature13241 24828044

[68] Calder PC. n-3 Polyunsaturated fatty acids, inflammation, and inflammatory. Am J Clin Nutr. 2006;83: 1505–1519. 10.1093/ajcn/83.6.1505S 16841861

[69] Rayhan RU, Stevens BW, Raksit MP, Ripple JA, Timbol CR, Adewuyi O, et al Exercise Challenge in Gulf War Illness Reveals Two Subgroups with Altered Brain Structure and Function. PLoS One. 2013;8. 10.1371/journal.pone.0063903 PMC3683000 23798990

[70] Gibellini F, Smith TK. The Kennedy pathway-De novo synthesis of phosphatidylethanolamine and phosphatidylcholine. IUBMB Life. 2010;62: n/a–n/a. 10.1002/iub.337 20503434

[71] Karen DJ, Li W, Harp PR, Gillette JS, Bloomquist JR. Striatal dopaminergic pathways as a target tor the insecticides permethrin and chlorpyrifos. Neurotoxicology. 2001;22: 811–817. 11829414 10.1016/s0161-813x(01)00063-8

[72] Hoy J. Repeated coadministrations of pyridostigmine bromide, DEET, and permethrin alter locomotor behavior of rats. Vet Hum Toxicol. 2000;42: 72–76. 10750169

[73] Chaney LM. Toxic interactions between pyridostigmine (PB), N,N-Diethyl-m-toluamide (DEET), adrenergic agents and caffeine. Toxicologist. 1997;106: 21.

[74] Flunker LK, Nutter TJ, Johnson RD, Cooper BY. DEET potentiates the development and persistence of anticholinesterase dependent chronic pain signs in a rat model of Gulf War Illness pain. Toxicol Appl Pharmacol. Elsevier Inc.; 2017;316: 48–62. 10.1016/j.taap.2016.12.014 28025109

