# Supplementary material for: Phospholipid profiling of plasma from GW veterans and rodent models to identify potential biomarkers of Gulf War Illness
Source: PLoS One. 2017 Apr 28;12(4):e0176634. doi: 10.1371/journal.pone.0176634 (PMC5409146; doi:10.1371/journal.pone.0176634)
Supplement: S1 Table — In humans, for PC 8 components were identified with PCA analysis, of which components 2 (p = 0.005), 4 (p<0.001), 5 (p = 0.006) and 6 (p = 0.032) were significant for GWI diagnosis. For LPC 8 components were identified of which component 3 was significant for GWI (p = 0.003). For PE, component 4 was significant (p = 0.014) out of 8 identified. For LPE, out of 4 identified components, component 1 was significant (p<0.001) for GWI. For PI only one component was identified and significant via PCA (p = 0.002). For SM, 3 components were identified with PCA analysis, of which component 1 was significant for GWI (p = 0.032). (DOCX) [file pone.0176634.s001.docx]

| **Component** | **2** | **4** | **5** | **6** | **3** | **4** | **1** | **1** | **1** |
| --- | --- | --- | --- | --- | --- | --- | --- | --- | --- |
| **% Total variance** | **15.81** | **6.18** | **4.7** | **4.31** | **8.55** | **11.89** | **46.32** | **75.37** | **57.44** |
| 1 |  |  |  |  | LPC(0-16:1) | ePE(40:3) | LPE(o-16:0) | PI(34:2) | DSM(16:0) |
| 2 |  |  | ePC(38:0) |  | LPC(0-18:0) | ePE(40:4) | LPE(16:1) | PI(34:1) | DSM(22:0) |
| 3 | ePC(38:3) |  |  |  | LPC(0-20:1) | ePE(40:5) | LPE(16:0) | PI(36:4) | SM(16:0) |
| 4 | ePC(38:4) |  |  |  | LPC(18:2) | PE(40:4) | LPE(18:2) | PI(36:3) | SM(16:1) |
| 5 | ePC(38:5) |  |  |  | LPC(18:3) | PE(42:10) | LPE(18:0) | PI(36:2) | SM(22:0) |
| 6 |  |  | ePC(38:6) |  | LPC(20:2) | PE(42:2) | LPE(o-20:1) | PI(38:5) | SM(22:1) |
| 7 |  |  | ePC(40:6) |  | LPC(22:5) | PE(42:3) | LPE(20:4) | PI(38:4) | SM(24:0) |
| 8 |  | PC(30:0) |  |  |  | PE(42:4) | LPE(22:6) | PI(40:6) | SM(24:1) |
| 9 | PC(32:0) | PC(32:0) |  |  |  | PE(42:6) | LPE(22:5) | PI(40:5) |  |
| 10 | PC(32:1) | PC(32:1) |  |  |  | PE(42:7) | LPE(22:1) |  |  |
| 11 |  | PC(32:2) |  |  |  | PE(42:8) |  |  |  |
| 12 | PC(34:0) |  |  | PC(34:0) |  | PE(42:9) |  |  |  |
| 13 | PC(34:1) | PC(34:1) |  |  |  | PE(44:12) |  |  |  |
| 14 |  |  |  | PC(34:2) |  | PE(44:6) |  |  |  |
| 15 | PC(34:3) | PC(34:3) |  | PC(34:3) |  | PE(44:7) |  |  |  |
| 16 |  | PC(34:4) |  |  |  | PE(44:8) |  |  |  |
| 17 | PC(36:1) |  |  |  |  | PE(44:9) |  |  |  |
| 18 |  |  |  | PC(36:2) |  |  |  |  |  |
| 19 | PC(36:3) |  |  | PC(36:3) |  |  |  |  |  |
| 20 | PC(36:4) |  |  |  |  |  |  |  |  |
| 21 |  | PC(36:5) | PC(36:5) |  |  |  |  |  |  |
| 22 | PC(38:3) |  |  |  |  |  |  |  |  |
| 23 | PC(38:4) |  |  |  |  |  |  |  |  |
| 24 | PC(38:5) |  | PC(38:5) |  |  |  |  |  |  |
| 25 |  |  | PC(38:6) |  |  |  |  |  |  |
| 26 | PC(40:4) |  |  |  |  |  |  |  |  |
| 27 | PC(40:5) |  |  |  |  |  |  |  |  |
| 28 | PC(40:6) |  | PC(40:6) |  |  |  |  |  |  |
| 29 |  |  | PC(40:7) |  |  |  |  |  |  |

**S1 Table**
