# Supplementary material for: Phospholipid profiling of plasma from GW veterans and rodent models to identify potential biomarkers of Gulf War Illness
Source: PLoS One. 2017 Apr 28;12(4):e0176634. doi: 10.1371/journal.pone.0176634 (PMC5409146; doi:10.1371/journal.pone.0176634)
Supplement: S2 Table — In mice, for PC 5 components were identified with PCA analysis, of which component 4 (p = 0.005) was significant for PB+PER exposure. For LPC 4 components were identified of which component 1 was significant for PB+PER exposure (p<0.001). For PE, component 1 was significant (p = 0.036) out of 5 identified. For LPE, out of 3 identified components, component 1 was significant (p = 0.009) for GW agent exposure. For PI 4 components were identified and significant via PCA for component 3 (p = 0.008). For SM, 3 components were identified with PCA analysis, of which component 3 was significant for PB+PER (p = 0.044). (DOCX) [file pone.0176634.s002.docx]

| **Component** | **4** | **1** | **1** | **1** | **3** |
| --- | --- | --- | --- | --- | --- |
| **% Total variance** | **5.12** | **39.78** | **66.05** | **49.37** | **10.92** |
| 1 | ePC(34:0) | LPC(16:0) | ePE(34:1) | LPE(16:0) | PI(34:1) |
| 2 | ePC(36:0) | LPC(16:1) | ePE(34:3) | LPE(18:0) | PI(34:2) |
| 3 | ePC(36:1) | LPC(18:0) | ePE(36:1) | LPE(18:2) | PI(36:1) |
| 4 | ePC(36:2) | LPC(18:1) | ePE(36:2) | LPE(20:4) | PI(36:2) |
| 5 | ePC(38:1) | LPC(18:2) | ePE(36:3) | LPE(22:6) | PI(36:3) |
| 6 | ePC(38:2) | LPC(20:3) | ePE(36:4) |  | PI(36:4) |
| 7 | ePC(38:3) | LPC(20:4) | ePE(36:5) |  | PI(37:4) |
| 8 | ePC(40:4) | LPC(22:6) | ePE(38:0) |  | PI(38:4) |
| 9 | PC(34:2) |  | ePE(38:1) |  | PI(38:6) |
| 10 | PC(36:6) |  | ePE(38:2) |  | PI(39:4) |
| 11 | PC(38:1) |  | ePE(38:3) |  | PI(40:4) |
| 12 |  |  | ePE(38:6) |  | PI(40:6) |
| 13 |  |  | ePE(40:4) |  | PI(42:10) |
| 14 |  |  | PE(36:0) |  |  |
| 15 |  |  | PE(36:2) |  |  |
| 16 |  |  | PE(36:4) |  |  |
| 17 |  |  | PE(38:3) |  |  |
| 18 |  |  | PE(40:3) |  |  |
| 19 |  |  | PE(40:5) |  |  |
| 20 |  |  | PE(40:7) |  |  |
| 21 |  |  | PE(42:4) |  |  |
| 22 |  |  | PE(42:5) |  |  |
| 23 |  |  | PE(42:6) |  |  |
| 24 |  |  | PE(42:7) |  |  |
| 25 |  |  | PE(42:8) |  |  |
| 26 |  |  | PE(44:10) |  |  |
| 27 |  |  | PE(44:11) |  |  |
| 28 |  |  | PE(44:12) |  |  |
| 29 |  |  | PE(44:7) |  |  |
| 30 |  |  | PE(44:8) |  |  |
| 31 |  |  | PE(44:9) |  |  |

**S2 Table**
