# Supplementary material for: Phospholipid profiling of plasma from GW veterans and rodent models to identify potential biomarkers of Gulf War Illness
Source: PLoS One. 2017 Apr 28;12(4):e0176634. doi: 10.1371/journal.pone.0176634 (PMC5409146; doi:10.1371/journal.pone.0176634)
Supplement: S3 Table — In rats, for PC 3 components were identified with PCA analysis, of which component 1 (p = 0.017) and 2 (p<0.001) were significant for PB+PER+DEET and stress exposure. For LPC only 1 component was identified and significant for PB+PER+DEET and stress exposure (p<0.001). For PE, components 2 (p = 0.003), 4 (p = 0.017), and 5 (p = 0.001) were significant out of 5 identified. For LPE, out of 3 identified components, component 2 was significant (p = 0.003) for GW agent and stress exposure. For PI 3 components were identified and significant via PCA for component 1 (p<0.001). For SM, 1 component was identified with PCA analysis and significant for PB+PER+DEET and stress exposure (p<0.001). (DOCX) [file pone.0176634.s003.docx]

| **Component** | **1** | **2** | **1** | **2** | **4** | **5** | **2** | **1** | **1** |
| --- | --- | --- | --- | --- | --- | --- | --- | --- | --- |
| **% Total variance** | **89.83** | **3.05** | **82.36** | **7.28** | **2.1** | **1.57** | **18.45** | **60.23** | **83.15** |
| 1 | ePC(34:0) | ePC(34:0) | LPC(0-16:0) |  | ePE(34:3) |  | LPE(20:3) | PI(34:1) | DSM(16:0) |
| 2 | ePC(34:1) | ePC(34:1) | LPC(0-18:0) |  | ePE(36:2) |  | LPE(o-16:1) | PI(34:2) | DSM(18:0) |
| 3 | ePC(34:2) | ePC(34:2) | LPC(0-20:0) |  | ePE(36:3) |  | LPE(o-20:1) | PI(36:1) | DSM(22:0) |
| 4 | ePC(36:0) | ePC(36:0) | LPC(0-20:1) | ePE(36:4) |  |  |  | PI(36:2) | DSM(24:0) |
| 5 | ePC(36:1) | ePC(36:1) | LPC(16:0) |  | ePE(38:2) |  |  | PI(36:3) | SM(16:0) |
| 6 | ePC(36:2) | ePC(36:2) | LPC(18:0) | ePE(40:3) |  |  |  | PI(36:4) | SM(16:1) |
| 7 | ePC(36:4) | ePC(36:4) | LPC(18:1) |  | ePE(40:4) |  |  | PI(36:6) | SM(18:0) |
| 8 | ePC(38:0) | ePC(38:0) | LPC(18:2) |  | ePE(40:5) |  |  | PI(37:4) | SM(18:1) |
| 9 | ePC(38:1) | ePC(38:1) | LPC(20:3) |  | ePE(40:6) |  |  | PI(38:0) | SM(22:0) |
| 10 | ePC(38:2) | ePC(38:2) | LPC(20:4) | PE(34:1) |  |  |  | PI(38:1) | SM(22:1) |
| 11 | ePC(38:3) | ePC(38:3) | LPC(22:5) | PE(34:2) |  | PE(34:2) |  | PI(38:2) | SM(24:0) |
| 12 | ePC(38:4) | ePC(38:4) | LPC(22:6) | PE(36:0) | PE(36:0) |  |  | PI(38:3) | SM(24:1) |
| 13 | ePC(38:5) | ePC(38:5) |  | PE(36:1) |  | PE(36:1) |  | PI(38:4) |  |
| 14 | ePC(38:6) | ePC(38:6) |  | PE(36:2) |  | PE(36:2) |  | PI(38:5) |  |
| 15 | ePC(40:2) | ePC(40:2) |  |  |  | PE(36:3) |  | PI(38:6) |  |
| 16 | ePC(40:3) | ePC(40:3) |  | PE(36:4) |  |  |  | PI(39:4) |  |
| 17 | ePC(40:4) | ePC(40:4) |  | PE(38:0) | PE(38:0) |  |  | PI(40:0) |  |
| 18 | ePC(40:5) | ePC(40:5) |  | PE(38:1) | PE(38:1) |  |  | PI(40:1) |  |
| 19 | ePC(40:6) | ePC(40:6) |  | PE(38:4) |  |  |  | PI(40:2) |  |
| 20 |  | PC(32:0) |  | PE(38:5) |  |  |  | PI(40:3) |  |
| 21 | PC(32:1) | PC(32:1) |  | PE(38:6) |  |  |  | PI(40:4) |  |
| 22 | PC(32:2) | PC(32:2) |  | PE(40:5) |  |  |  | PI(40:5) |  |
| 23 | PC(34:0) | PC(34:0) |  | PE(40:6) |  |  |  | PI(40:6) |  |
| 24 | PC(34:1) | PC(34:1) |  | PE(40:7) |  |  |  | PI(40:7) |  |
| 25 | PC(34:2) | PC(34:2) |  | PE(42:3) |  |  |  | PI(42:10) |  |
| 26 | PC(34:3) | PC(34:3) |  | PE(42:4) |  |  |  | PI(42:2) |  |
| 27 | PC(36:1) | PC(36:1) |  |  |  |  |  | PI(42:3) |  |
| 28 | PC(36:2) | PC(36:2) |  |  |  |  |  | PI(42:4) |  |
| 29 | PC(36:3) | PC(36:3) |  |  |  |  |  | PI(42:5) |  |
| 30 | PC(36:4) | PC(36:4) |  |  |  |  |  | PI(42:7) |  |
| 31 | PC(36:5) | PC(36:5) |  |  |  |  |  |  |  |
| 32 | PC(36:6) | PC(36:6) |  |  |  |  |  |  |  |
| 33 | PC(38:0) | PC(38:0) |  |  |  |  |  |  |  |
| 34 | PC(38:1) | PC(38:1) |  |  |  |  |  |  |  |
| 35 | PC(38:3) | PC(38:3) |  |  |  |  |  |  |  |
| 36 | PC(38:4) | PC(38:4) |  |  |  |  |  |  |  |
| 37 | PC(38:5) | PC(38:5) |  |  |  |  |  |  |  |
| 38 | PC(38:6) | PC(38:6) |  |  |  |  |  |  |  |
| 39 | PC(40:2) | PC(40:2) |  |  |  |  |  |  |  |
| 40 | PC(40:3) | PC(40:3) |  |  |  |  |  |  |  |
| 41 | PC(40:4) | PC(40:4) |  |  |  |  |  |  |  |
| 42 | PC(40:5) | PC(40:5) |  |  |  |  |  |  |  |
| 43 | PC(40:6) | PC(40:6) |  |  |  |  |  |  |  |
| 44 | PC(40:7) | PC(40:7) |  |  |  |  |  |  |  |
| 45 | PC(40:8) | PC(40:8) |  |  |  |  |  |  |  |
| 46 | PC(42:10) | PC(42:10) |  |  |  |  |  |  |  |
| 47 | PC(42:11) | PC(42:11) |  |  |  |  |  |  |  |
| 48 | PC(42:3) | PC(42:3) |  |  |  |  |  |  |  |
| 49 | PC(42:6) |  |  |  |  |  |  |  |  |
| 50 | PC(42:7) | PC(42:7) |  |  |  |  |  |  |  |
| 51 | PC(42:8) | PC(42:8) |  |  |  |  |  |  |  |
| 52 | PC(42:9) | PC(42:9) |  |  |  |  |  |  |  |
| 53 | PC(44:12) | PC(44:12) |  |  |  |  |  |  |  |

**S3 Table**
