# Supplementary material for: Phospholipid profiling of plasma from GW veterans and rodent models to identify potential biomarkers of Gulf War Illness
Source: PLoS One. 2017 Apr 28;12(4):e0176634. doi: 10.1371/journal.pone.0176634 (PMC5409146; doi:10.1371/journal.pone.0176634)
Supplement: S4 Table — Individual molecular species of each class were quantified by LC/MS and summed after lipidomeDB analyses to generate total lipid levels. *denotes significant p values for p<0.05. (DOCX) [file pone.0176634.s004.docx]

|  | **Gulf War Veterans** | | | | | | **Mouse model** | | | | | | **Rat model** | | | | | |
| --- | --- | --- | --- | --- | --- | --- | --- | --- | --- | --- | --- | --- | --- | --- | --- | --- | --- | --- |
|  | **Control** | | | **GWI** | | | **Control** | | | **PB+PER** | | | **Control** | | | **PB+PER+DEET+Stress** | | |
| **total PC** | 1034.06 | ± | 22.40 | 1029.94 | ± | 32.73 | 2214.36 | ± | 74.17 | 2252.12 | ± | 131.01 | 553.92 | ± | 26.83 | 1276.48 | ± | 66.14* |
| **total LPC** | 272.84 | ± | 16.08 | 314.55 | ± | 16.08* | 78.52 | ± | 2.29 | 95.38 | ± | 3.21* | 445.64 | ± | 26.92 | 716.79 | ± | 30.78* |
| **total PE** | 89.60 | ± | 4.07 | 94.80 | ± | 3.77 | 117.60 | ± | 5.56 | 114.23 | ± | 6.39 | 45.69 | ± | 1.54 | 65.26 | ± | 2.57* |
| **total LPE** | 14.42 | ± | 0.75 | 15.57 | ± | 0.60 | 54.56 | ± | 1.51 | 61.18 | ± | 3.04* | 6.70 | ± | 0.14 | 6.35 | ± | 0.15 |
| **total PI** | 87.39 | ± | 3.53 | 72.69 | ± | 2.71 | 234.63 | ± | 6.10 | 249.49 | ± | 7.48 | 90.21 | ± | 4.48 | 216.07 | ± | 9.78* |
| **total SM** | 227.69 | ± | 4.87 | 220.29 | ± | 8.09 | 71.66 | ± | 1.85 | 74.56 | ± | 2.11 | 64.32 | ± | 3.64 | 118.44 | ± | 4.87 |

**S4 Table**
