# Supplementary material for: Phospholipid profiling of plasma from GW veterans and rodent models to identify potential biomarkers of Gulf War Illness
Source: PLoS One. 2017 Apr 28;12(4):e0176634. doi: 10.1371/journal.pone.0176634 (PMC5409146; doi:10.1371/journal.pone.0176634)
Supplement: S5 Table — *denotes significant p values for p<0.05. (DOCX) [file pone.0176634.s005.docx]

|  |  | **Gulf War Veteran** | | | | | | **Mouse model** | | | | | | **Rat model** | | | | | |
| --- | --- | --- | --- | --- | --- | --- | --- | --- | --- | --- | --- | --- | --- | --- | --- | --- | --- | --- | --- |
|  |  | **Control** | | | **PB+PER** | | | **Control** | | | **PB+PER** | | | **Control** | | | **PB+PER+DEET+Stress** | | |
| **PC** | *SFA* | 32.15 | ± | 1.19 | 28.37 | ± | 1.09* | 41.70 | ± | 1.85 | 41.47 | ± | 3.05 | 23.68 | ± | 0.52 | 51.97 | ± | 2.28* |
|  | *MUFA* | 143.14 | ± | 6.42 | 135.43 | ± | 5.90 | 179.83 | ± | 9.08 | 218.41 | ± | 23.04 | 49.11 | ± | 1.98 | 109.30 | ± | 6.00* |
|  | *PUFA* | 474.41 | ± | 12.91 | 506.87 | ± | 17.23 | 1102.12 | ± | 43.59 | 1073.86 | ± | 67.04 | 298.28 | ± | 17.86 | 706.53 | ± | 45.01* |
| **LPC** | *SFA* | 182.88 | ± | 12.38 | 211.50 | ± | 12.26* | 45.31 | ± | 1.41 | 52.51 | ± | 1.61* | 270.48 | ± | 17.29 | 399.15 | ± | 17.95* |
|  | *MUFA* | 34.74 | ± | 1.98 | 39.89 | ± | 1.95* | 8.17 | ± | 0.27 | 11.06 | ± | 0.84* | 45.10 | ± | 2.09 | 77.95 | ± | 2.91* |
|  | *PUFA* | 16.96 | ± | 1.20 | 20.91 | ± | 1.17* | 9.45 | ± | 0.39 | 12.02 | ± | 0.43* | 130.06 | ± | 7.87 | 239.68 | ± | 11.25* |
| **PE** | *SFA* | 22.64 | ± | 1.02 | 22.31 | ± | 0.99 | 8.26 | ± | 0.48 | 7.77 | ± | 0.37* | 2.55 | ± | 0.15 | 4.12 | ± | 0.19* |
|  | *MUFA* | 5.75 | ± | 0.23 | 5.74 | ± | 0.22 | 12.69 | ± | 0.41 | 13.19 | ± | 0.92 | 5.46 | ± | 0.19 | 7.02 | ± | 0.19* |
|  | *PUFA* | 54.15 | ± | 3.30 | 59.08 | ± | 2.76 | 89.23 | ± | 4.47 | 85.44 | ± | 4.64* | 30.83 | ± | 1.04 | 44.10 | ± | 2.02* |
| **LPE** | *SFA* | 3.90 | ± | 0.15 | 3.75 | ± | 0.21 | 16.32 | ± | 0.78 | 17.15 | ± | 0.78 | 0.22 | ± | 0.03 | 0.11 | ± | 0.05* |
|  | *MUFA* | 5.78 | ± | 0.22 | 5.40 | ± | 0.17 | 11.49 | ± | 0.49 | 12.07 | ± | 0.79 | 6.19 | ± | 0.11 | 5.95 | ± | 0.12 |
|  | *PUFA* | 2.71 | ± | 0.34 | 4.06 | ± | 0.21* | 17.73 | ± | 0.57 | 20.65 | ± | 1.25 | 0.24 | ± | 0.03 | 0.26 | ± | 0.05 |
| **PI** | *SFA* | 2.18 | ± | 0.29 | 0.71 | ± | 0.06 | 0.38 | ± | 0.05 | 0.44 | ± | 0.06 | 0.68 | ± | 0.08 | 0.88 | ± | 0.05* |
|  | *MUFA* | 24.93 | ± | 3.08 | 9.07 | ± | 0.44 | 4.60 | ± | 0.23 | 5.22 | ± | 0.26 | 24.93 | ± | 3.08 | 9.07 | ± | 0.44* |
|  | *PUFA* | 48.55 | ± | 2.02 | 51.40 | ± | 2.06 | 202.70 | ± | 5.01 | 214.55 | ± | 7.00 | 78.37 | ± | 4.16 | 189.28 | ± | 8.92* |

**S5 Table**
