# Supplementary material for: Phospholipid profiling of plasma from GW veterans and rodent models to identify potential biomarkers of Gulf War Illness
Source: PLoS One. 2017 Apr 28;12(4):e0176634. doi: 10.1371/journal.pone.0176634 (PMC5409146; doi:10.1371/journal.pone.0176634)
Supplement: S6 Table — *denotes significant p values for p<0.05. (DOCX) [file pone.0176634.s006.docx]

|  | **Gulf War Veteran** | | | | | | **Mouse model** | | | | | | **Rat model** | | | | | |
| --- | --- | --- | --- | --- | --- | --- | --- | --- | --- | --- | --- | --- | --- | --- | --- | --- | --- | --- |
|  | **Control** | | | **PB+PER** | | | **Control** | | | **PB+PER** | | | **Control** | | | **PB+PER+DEET+Stress** | | |
| **ePC** | 99.79 | ± | 3.31 | 97.07 | ± | 3.78 | 90.07 | ± | 4.48 | 87.19 | ± | 8.01 | 66.63 | ± | 3.58 | 150.84 | ± | 7.94* |
| **eLPC** | 11.12 | ± | 0.58 | 12.45 | ± | 0.62 | 1.97 | ± | 0.10 | 1.98 | ± | 0.14 | 25.36 | ± | 1.11 | 36.77 | ± | 1.02* |
| **ePE** | 37.71 | ± | 2.10 | 40.43 | ± | 2.10 | 48.63 | ± | 1.97 | 46.96 | ± | 2.07 | 15.15 | ± | 1.09 | 19.65 | ± | 0.74* |
| **eLPE** | 2.94 | ± | 0.30 | 4.20 | ± | 0.15* | 9.64 | ± | 0.45 | 10.11 | ± | 0.72 | 6.19 | ± | 0.11 | 5.95 | ± | 0.12 |

**S6 Table**
