# Supplementary material for: Phospholipid profiling of plasma from GW veterans and rodent models to identify potential biomarkers of Gulf War Illness
Source: PLoS One. 2017 Apr 28;12(4):e0176634. doi: 10.1371/journal.pone.0176634 (PMC5409146; doi:10.1371/journal.pone.0176634)
Supplement: S7 Table — No DHA containing LPE species were identified for the rat model, therefore ratio for AAtoDHA containing LPE species could not be determined.*denotes significant p values for p<0.05. (DOCX) [file pone.0176634.s007.docx]

|  |  | **Gulf War Veteran** | | | | | | **Mouse model** | | | | | | **Rat model** | | | | | |
| --- | --- | --- | --- | --- | --- | --- | --- | --- | --- | --- | --- | --- | --- | --- | --- | --- | --- | --- | --- |
|  |  | **Control** | | | **PB+PER** | | | **Control** | | | **PB+PER** | | | **Control** | | | **PB+PER+DEET+Stress** | | |
| **PC** | *DHA* | 59.97 | ± | 2.63 | 71.03 | ± | 3.03* | 282.86 | ± | 12.82 | 252.75 | ± | 16.20 | 52.03 | ± | 3.51 | 143.08 | ± | 13.85* |
|  | *AA* | 234.08 | ± | 9.42 | 253.26 | ± | 10.04 | 570.28 | ± | 23.42 | 554.00 | ± | 30.82 | 184.04 | ± | 11.11 | 414.24 | ± | 23.47* |
| **LPC** | *DHA* | 1.86 | ± | 0.13 | 2.64 | ± | 0.17* | 2.23 | ± | 0.12 | 2.58 | ± | 0.18 | 8.07 | ± | 0.89 | 18.63 | ± | 1.89* |
|  | *AA* | 10.32 | ± | 0.76 | 12.64 | ± | 0.75* | 6.00 | ± | 0.23 | 7.70 | ± | 0.28* | 52.81 | ± | 3.58 | 95.18 | ± | 5.95* |
| **PE** | *DHA* | 3.34 | ± | 0.20 | 3.82 | ± | 0.18 | 9.21 | ± | 0.52 | 8.23 | ± | 0.37 | 1.81 | ± | 0.09 | 3.58 | ± | 0.27* |
|  | *AA* | 10.22 | ± | 0.84 | 10.90 | ± | 0.62 | 18.80 | ± | 0.97 | 17.73 | ± | 0.99 | 7.14 | ± | 0.40 | 11.48 | ± | 0.81* |
| **LPE** | *DHA* | 0.88 | ± | 0.12 | 1.41 | ± | 0.10* | 8.65 | ± | 0.25 | 10.09 | ± | 0.70 | N/A |  |  |  |  |  |
|  | *AA* | 1.38 | ± | 0.19 | 1.94 | ± | 0.10* | 8.14 | ± | 0.28 | 9.75 | ± | 0.70* | 0.07 | ± | 0.02 | 0.10 | ± | 0.03 |
| **PI** | *DHA* | 1.21 | ± | 0.06 | 1.35 | ± | 0.08 | 3.43 | ± | 0.09 | 3.53 | ± | 0.10 | 2.48 | ± | 0.19 | 7.11 | ± | 0.70* |
|  | *AA* | 29.55 | ± | 1.76 | 35.13 | ± | 1.43 | 139.41 | ± | 3.06 | 143.04 | ± | 4.21 | 54.34 | ± | 3.35 | 133.51 | ± | 6.27* |

**S7 Table**
